# On the nature of high-spin forms in the S_2_ state of the oxygen-evolving complex†

**DOI:** 10.1039/d4sc07818g

**Published:** 2025-01-31

**Authors:** Markella Aliki Mermigki, Maria Drosou, Dimitrios A. Pantazis

**Affiliations:** a Max-Planck-Institut für Kohlenforschung Kaiser-Wilhelm-Platz 1 45470 Mülheim an der Ruhr Germany dimitrios.pantazis@kofo.mpg.de

## Abstract

The Mn_4_CaO_*x*_ cluster of the oxygen-evolving complex (OEC) in photosystem II, the site of biological water oxidation, adopts different forms as it progresses through the catalytic cycle of S_*i*_ states (*i* = 0–4) and within each S_*i*_ state itself. This has been amply documented by spectroscopy, but the structural basis of spectroscopic polymorphism remains debated. The S_2_ state is extensively studied by magnetic resonance spectroscopies. In addition to the common type of *g* ≈ 2 multiline EPR signal attributed to a low-spin (*S* = 1/2) form of the manganese cluster, other signals at lower fields (*g* ≥ 4) associated with the S_2_ state arise from higher-spin forms. Resolving the structural identity of the high-spin species is paramount for a microscopic understanding of the catalytic mechanism. Hypotheses explored by theoretical studies implicate valence isomerism, proton tautomerism, or coordination change with respect to the low-spin form. Here we analyze structure–property correlations for multiple formulations employing a common high-level protocol based on multiscale models that combine a converged quantum mechanics region embedded within a large protein region treated semiempirically with an extended tight-binding method (DFT/xTB), surpassing conventional quantum mechanics/molecular mechanics (QM/MM) approaches. Our results provide a comprehensive comparison of magnetic topologies, spin states and energetics in relation to experimental observations. Crucial predictions are made about ^14^N hyperfine coupling constants and X-ray absorption Mn K-pre-edge features as criteria for discriminating between different models. This study updates our view on a persistent mystery of biological water oxidation, while providing a refined and transferable computational platform for future theoretical studies of the OEC.

## Abstract 2

Candidate models for the high-spin forms of the S_2_ state of the oxygen-evolving complex are evaluated using multilayer DFT/xTB simulations, explaining how their magnetic, spectroscopic, and energetic properties align with experimental observations.

## Graphical abstract

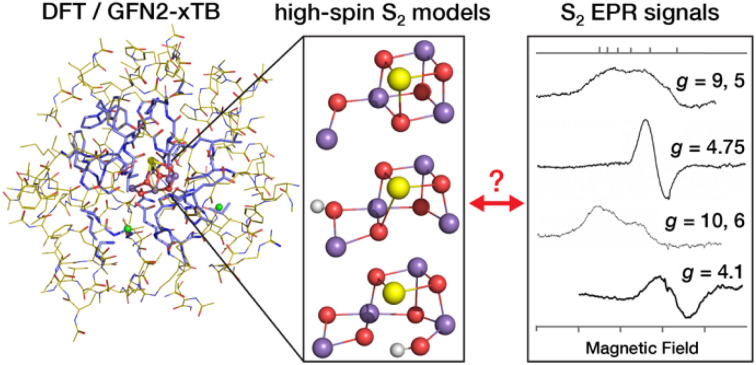

## 1. Introduction

**Fig. 1:**
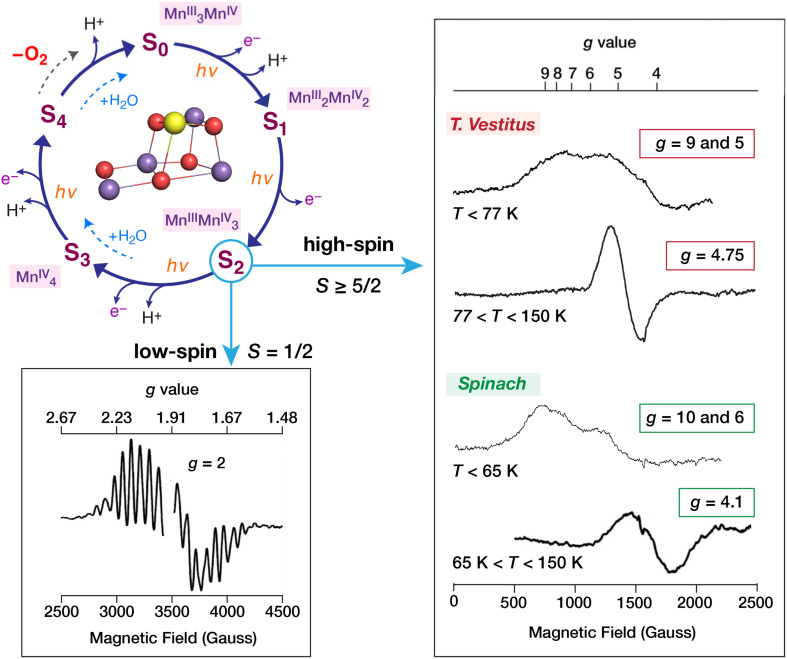
S-state cycle of the OEC involving sequential one-electron oxidations by the proximal tyrosyl radical 
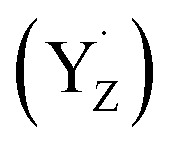
, proton release, and substrate water binding. X-band EPR signals observed for the S_2_ state under native conditions: the *g* ≈ 2 multiline signal attributed to a low-spin *S* = 1/2 ground state (adapted with permission from ref. 17), and four EPR signals observed at higher *g* values and attributed to high-spin forms with *S* ≥ 5/2: in spinach at *g* = 4.1 (adapted with permission from ref. 17) and at *g* = 10 and *g* = 6 (adapted with permission from ref. 18, Copyright 2024 American Chemical Society), and in *T. vestitus* at *g* = 4.75 (adapted from ref. 19 with permission from Elsevier) and at *g* = 9 and *g* = 5 (adapted with permission from ref. 20, Copyright 2024 American Chemical Society). The signals have been adapted for purposes of presentation.

The oxygen-evolving complex (OEC) of photosystem II (PSII) drives oxygenic photosynthesis by catalyzing the four-electron oxidation of water to dioxygen.^1–3^ The active site contains a Mn_4_CaO_*x*_ (*x* = 5 or 6) cluster bound to the protein matrix mostly *via* carboxylate residues.^4^ It is located in the transmembrane region of PSII, therefore water ingress and proton egress are mediated by specific channels and hydrogen-bonding networks.^5–7^ The OEC traverses a series of intermediate states S_*i*_ (*i* = 0–4)^8^ as it becomes oxidatively charged through successive abstraction of electrons^9–12^ (Fig. 1) by a highly oxidizing chlorophyll-centered cation that is created by light-driven charge separation at the reaction center of PSII.^13–16^ Electron transfer from the Mn cluster to the reaction center is mediated by a proximal tyrosine residue that cycles between the neutral protonated and the neutral radical (tyrosyl) form which serves as the immediate oxidant of the metal cluster.

Among the astounding diversity of experimental approaches employed in the study of the OEC, electron paramagnetic resonance (EPR) spectroscopy has a special place owing to its ability to report directly and selectively on the electronic structure of the electronically open-shell cluster.^2,21–27^ The S_2_ state, formed by one-electron oxidation of the “dark-stable” S_1_ state, is of particular importance for understanding the mechanism of biological water oxidation because it leads the system to the final metastable state of the cycle, S_3_,^28–33^ which prepares the catalyst for O–O bond formation and O_2_ evolution.^34–37^ The S_2_ state has been a target of historical significance in EPR studies of the OEC. Its characteristic multiline EPR signal at *g* ≈ 2 (with 18 or more distinguishable lines)^38–47^ first revealed the nature of the OEC as a cluster of closely interacting Mn ions, magnetically coupled to a state of total spin *S* = 1/2. The S_2_ state has been an anchor point for magnetic resonance studies of the OEC ever since.^17,24^

Modified versions of the multiline *g* ≈ 2 signal arise under different conditions, such as treatment of the S_2_ samples with ammonia,^48–50^ substitution of Ca^2+^ for Sr^2+^,^51–54^ and Ca^2+^ depletion.^52,55^ The various recorded *g* ≈ 2 multiline signals feature similar hyperfine structures and slightly variable splitting and total width, depending on the organisms, sample preparation, and chemical modifications. These variations are typically attributed to small perturbations of the electronic structure of the Mn_4_CaO_5_ cluster.

A central result of EPR spectroscopy is the presence of multiple signals in the various observable S_*i*_ states of the OEC.^23^ This is prominently the case in the S_2_ state, for which in addition to the *g* ≈ 2 multiline (“low-spin”) signal, many other signals have been observed at lower field (higher *g*) values.^18–20,56^ These higher-*g* signals are typically featureless and are collectively referred to as “high-spin” signals because they are attributed to forms of the Mn cluster with total spin *S* ≥ 3/2. This family of signals includes a multitude of resonances that appear in a wide range of *g* values, approximately from 4 to 10.

Distinct high-spin signals appear in different preparations and under different treatments (Fig. 1). Importantly, plants and cyanobacteria display different high-spin signals.^20^ In the case of spinach, as representative of higher-plant PSII, alongside the multiline low-spin signal there is a signal around *g* ≈ 4.1–4.25,^56–58^ depending on preparation conditions, attributed to a *S* = 5/2 state.^59^ This could correspond to even half of the S_2_ population in spinach under usual conditions for S_1_ → S_2_ progression.^40^ The low-spin and high-spin forms can be preferentially prepared by illumination of the S_1_ state at appropriate temperatures. Moreover, the state responsible for the multiline signal is converted to the state giving the *g* ≈ 4.1 signal by near-infrared radiation at a temperature lower than 150 K.^60^ An additional signal with resonances at *g* ≈ 6 and *g* ≈ 10, attributed to a *S* ≥ 5/2 state, is generated after near-infrared illumination at lower temperature *T* < 65 K.^18^ Diverging from the high-plant phenomenology, the *g* ≈ 4.1 signal is not natively observed in cyanobacteria, but a *g* ≈ 4.75, signal assigned to a *S* = 7/2 state can be induced by briefly warming the *g* ≈ 2 state at high pH (>6.5, p*K*_a_ 8.6).^19^ In analogy to spinach, near-infrared illumination (*T* < 77 K) in cyanobacteria induces a signal with resonances at *g* ≈ 5.5 and *g* ≈ 8.5.^20^ These high-*g* signals convert to the *g* ≈ 2 multiline signal at higher temperatures, suggesting that the low-spin and high-spin forms are interconvertible under physiological conditions. Enzyme modifications such as depletion or substitution of Cl^−^ or Ca^2+^ ions,^51,60–66^ depletion of extrinsic proteins,^67–69^ mutations,^70–77^ and treatments with small molecules able to bind near or directly on the OEC^48,78–92^ shift the equilibrium between the low-spin and high-spin S_2_ forms or lead to signals at different resonance positions.^17,23^

Low-field S_2_ EPR signals originate from energetically accessible high-spin states of the tetramanganese cluster that can be stabilized or even become more favorable than the low-spin *g* ≈ 2 state, even by minor perturbations. This is not only of fundamental interest but also has implications for the mechanism of water oxidation. This is because experimental studies suggest that high-spin S_2_ forms are directly involved in catalytic progression to the S_3_ state that prepares the cluster for O–O bond formation.^19,93^ Specifically, EPR experiments in *T. vestitus* showed that S_2_ samples were able to advance to the S_3_ state if they contained either only the *g* = 4.75 S_2_ state form or both the *g* = 4.75 and the low-spin *g* = 2 forms.^19^ Furthermore, based on the observation that a *g* ≈ 5 EPR signal, that resembles the *g* = 4.75 S_2_ signal, arises by near-infrared illumination (at 50 K) or prolonged incubation (at 77 K) of the S_3_ state,^94–98^ this form has been associated with an intermediate of the S_2_ → S_3_ catalytic transition.^19,93^

**Fig. 2:**
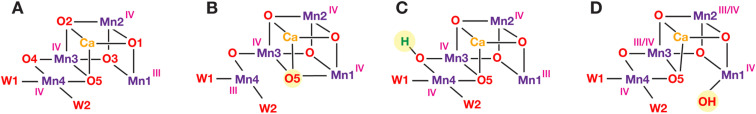
Proposed structural models that represent the S_2_ state in the low-spin form (A) and in high-spin forms: (B) valence isomerism; (C) oxo bridge protonation; (D) H_2_O/OH binding. Mn oxidation states proposed from theoretical calculations^99,101,102^ are also shown (in pink). For model D, two valence isomers have been reported,^101^ with the unique Mn(iii) ion in either the Mn2 or the Mn3 positions.

In terms of actual molecular geometries, combined contributions from crystallography, spectroscopy and quantum chemistry have largely converged to a specific structural model for the low-spin form of the S_2_ state that is associated with the *g* ≈ 2 signal(s). We coined the term “open cubane” to describe this form of the cluster (Fig. 2, form A) when we first reported its magnetic topology, spin state, correspondence with the *g* ≈ 2 signal and ^55^Mn hyperfine coupling constants compared to ^55^Mn electron-nuclear double resonance (ENDOR) spectroscopy.^99^ This structural model represents an oxo-bridged cluster with Mn oxidation states of iii–iv–iv–iv, where the O5 bridge that is positioned centrally in the cluster is closer to the terminal Mn4(iv) rather than the pseudo-Jahn–Teller axially distorted Mn1(iii). This creates a structural asymmetry and the impression that what could be a proper Mn_3_CaO_4_ cubane of Mn1, Mn2, and Mn3 ions has been “opened” along the Mn1–O5 vector. This topology, by enhancing superexchange interactions, is precisely the reason why the cluster in the S_2_ state has a low-spin ground state of *S* = 1/2.^99,100^

The situation for the high-spin forms is considerably less clear. EPR observations themselves cannot reveal the precise atomic structure behind these signals, while structural methods have not yet been successful at selectively characterizing high-spin forms of the S_2_ state. EXAFS data associated with the *g* ≈ 4.1 EPR signal in spinach have been reported,^101,103,104^ but have received conflicting interpretations, and at this point they cannot provide a conclusive geometric identity. Nevertheless, structure–property connections can be investigated with spectroscopy-oriented quantum chemistry.^105–108^ Plausible hypotheses about the structural origin of high-spin states have been mostly examined by quantum chemical studies that explore relationships between atomic structure and magnetic properties of the exchange-coupled system, the total spin being one point of contact with experimental observations.

Three main ideas exist about how to obtain a high-spin configuration of the manganese cluster in the S_2_ state. The first idea was suggested by our group in tandem with the explanation of the properties of the low-spin conformation: it involved a form of electronic isomerism that can be termed valence tautomerism, or valence exchange between the terminal Mn ions of the cluster, Mn1 and Mn4.^99^ In this scenario an intramolecular electron transfer/redox exchange occurs from Mn1(iii) to Mn4(iv) of the low-spin “open cubane” conformation. The valence rearrangement to Mn1(iv)–Mn4(iii) changes the magnetic exchange coupling pathways in such a way that ferromagnetic interactions are enhanced, leading to a higher total spin of *S* = 5/2 or 7/2. The fact that the geometry optimization of this high-spin valence tautomer leads to a shift of the O5 bridge closer to Mn1 than to Mn4 led us to call this isomer a “closed cubane” (Fig. 2, form B). These two forms are also referred to as “right/left elongated” by Isobe *et al.*^109^ Another idea was proposed by Corry and O'Malley,^102^ who showed that protonation of the O4 bridge in the low-spin “open cubane” conformation (Fig. 2, form C), a chemical change known to attenuate superexchange,^110–112^ can cause a switch of the total spin to higher values. This represents a minimal perturbation of the cluster geometry compared to the low-spin form. A third idea considers the possibility of coordination of an additional H_2_O or OH group.^101,113^ This scenario is also known as “early water binding” and has received support from substrate water exchange experiments.^114,115^ This involves the most extensive changes because it posits binding of a water-derived hydroxy ligand to Mn1 of the low-spin form, possibly accompanied by valence reorganization (Fig. 2, form D). This reconfigures the exchange coupling topology in a way that yields a high value for the total spin of the ground state. All three ideas, *i.e.*, valence isomerism through metal–metal electron transfer, change in bridge protonation states, or change in Mn coordination, involve modification of exchange coupling pathways compared to the low-spin form. Importantly, computational studies have suggested gating roles of these high-spin forms of the cluster in temporal and spatial control of the multistage S_2_ → S_3_ transition.^32,101,116–122^ Nevertheless, none of the three hypotheses has direct experimental support.

In an effort to advance the state of research with respect to the structural origins of the high-spin signals of the S_2_ state, here we create multiple variants of the currently discussed ideas and study their magnetic and spectroscopic properties. Our first goal is to describe the geometric structures of these models as accurately as possible. Toward this aim, we first define a new multilevel approach that combines DFT with an extended semiempirical tight-binding model (xTB).^123^ This resembles conventional quantum mechanics/molecular mechanics (QM/MM) approaches but has the advantage that a large part of the protein surrounding the high-level QM region can be simultaneously optimized using xTB instead of a force field, the description of the “low-level” region does not rely on fixed parameters dictated by the atom types of a classical force field, chemical events (including proton transfer) are allowed in the “low-level” region, and the treatment of the united system with xTB ensures explicit and full treatment of mutual polarization at this level. Magnetic, energetic, and spectroscopic properties are obtained *via* established protocols applied here in a multi-layer context. By carefully establishing convergence for both geometric structures and spectroscopic properties, we provide the most elaborate and methodologically advanced study so far on the subject, produce the “best case” models for each of the three high-spin ideas, present new models for each motif, and delineate future directions for both experimental and theoretical studies on the mechanism of biological water oxidation.

## 2. Methodology

### 2.1. Construction of models

**Fig. 3:**
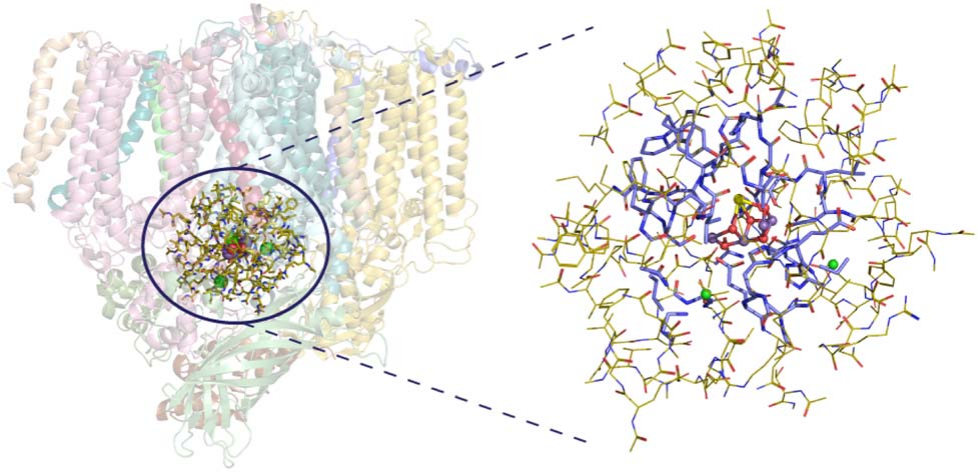
Representation of a PSII monomer as determined in the XFEL model of the S_2_ state by Kern *et al.* (PDB ID 6DHF, monomer A).^124^ The circle indicates the region included in the OEC model used in this work (2065 atoms). On the right, the OEC model is shown, with the region allowed to relax during geometry optimization depicted in dark purple sticks and the surrounding residues that were kept constrained shown in yellow. Hydrogen atoms are omitted for clarity.

The computational model of the OEC was constructed from the crystallographic coordinates of the XFEL structure of the 1F state of *T. vestitus* (formerly *T. elongatus*) PSII at 2.08 Å resolution (PDB: 6DHF, monomer A).^124^ This particular structure was selected as the optimal starting point because we recently determined it to be the most consistent with spectroscopic data on the S_2_ state.^125^ The model includes all amino acid residues and water molecules within 12 Å from the Mn_4_CaO_5_ cluster (Fig. 3), except from the chlorophyll *a* residue CLA405, which was excluded because it mostly protrudes from this region and in order to avoid potential issues arising from charge delocalization. Peptide chain ends at the boundary of the model were capped with neutral acetyl or *N*-methyl amide groups. In addition to the inorganic core Mn_4_CaO_5_ with its terminal water molecules W1–W4, the complete model includes in total the following amino acids, with residues whose backbone atoms were used for creating capping groups given in parentheses:

• D1 (chain A): (Pro57), Val58, Asp59, Ile60, Asp61, Gly62, Ile63, Arg64, Glu65, Pro66, Val67, (Ser68), (Val82), Val83, Pro84, Ser85, Ser86, Asn87, (Ala88), Ile89, Gly90, Leu91, (His92), (Pro111), Tyr112, (Gln113), (Ala156), Val157, (Phe158), (Leu159), Ile160, Tyr161, Pro162, Ile163, Gly164, Gln165, Gly166, Ser167, Phe168, Ser169, Asp170, Gly171, Met172, Pro173, (Leu174), (Ser177), Gly178, Thr179, Phe180, Asn181, Phe182, Met183, Ile184, Val185, Phe186, Gln187, Ala188, Glu189, His190, Asn191, Ile192, (Leu193), Glu289, Ile290, (Ser291), Thr292, Met293, Ala294, Phe295, Asn296, (Leu297), Asn298, (Gly299), (Ala324), Asn325, Leu326, Glu327, Met328, Glu329, Val330, Met331, His332, Glu333, Arg334, Asn335, Ala336, His337, Asn338, Phe339, Pro340, Leu341, Asp342, Leu343, Ala344.

• CP47 (chain B): Arg348.

• CP43 (chain C): (Val290), Trp291, (Phe292), (Thr305), Gry306, Pro307, Glu308, Ala309, Ser310, Gln311, Ala312, Gln313, Ala314, (Met315), (Pro334), Thr335, Glu354, Thr355, Met356, Arg357, Phe358, (Trp359), (His398), Ala399, Pro400, Leu401, (Gly402), (Gly408), Gly409, Val410, Ala411, Thr412, (Glu413).

• D2 (chain D): (Phe311), Glu312, Thr313, Phe314, (Tyr315), (Thr316), Lys317, Asn318, (Leu319), (Leu320), Leu321, (Asn322), (Arg348), Gly349, Asn350, Ala351, Leu352.

• PsbV: (Gly133), Lys134, (Val135).

Moreover, the model contains the two chloride ions and 50 water molecules. Several crystallographic models are known to exhibit lower water counts than those expected for a fully hydrated PSII.^5^ The 6DHF monomer A structure has 48 water molecules within 12 Å from the Mn_4_CaO_5_ cluster, which is a rather high number. The X-ray structure of the dark-adapted state of PSII at 1.85 Å resolution reported by Tanaka *et al.*^126^ (PDB: 5B66, monomer A) has the highest number of resolved water molecules overall,^5^ with 50 water molecules within 12 Å from the Mn_4_CaO_5_ cluster. The two additional water molecules from 5B66 were added to our 6DHF-based model; these were W671, located 4.0 Å from Mn3 in the O4 water channel, and W634, located 4.1 Å from Mn1 in the O1 water channel (labels according to 5B66 (ref. 126)). We note that the recently reported cryo-EM PSII model (9EVX)^6^ presents four additional candidate water positions in close proximity to the OEC, and within the largest radius from the OEC considered for optimization in the present study: W506 (occ. 0.31) and W619 (occ. 0.28) are near W4, with distances 2.3 and 2.0 Å, respectively, W532 (occ. 0.47) is near O1 with distance 2.0 Å from the nearest water molecule (W524), and W507 (occ. 0.45) is near Ala344 with distance 2.3 Å. Both the low estimated occupancies and the unphysically small distances from the nearest hydrogen bonding partners suggest that the first three positions cannot represent additional water molecules, but likely alternative positions of existing waters or other types of structure, potentially related to side-effects of cluster reduction.^6^ According to our simulations, only W507 might be spatially accommodated in the protein matrix of our computational model and may thus represent a real additional water molecule. However, given the uncertainty regarding its presence and the fact that it would not affect target properties studied in this work, we decided to not include it at present. All Asp and Glu residues were modeled as anions. All Arg residues (D1-Arg64, D1-Arg334, CP43-Arg357, and CP47-Arg348), all Lys residues (D2-Lys317, PsbV-Lys134) and D1-His337 were modeled as protonated cations.

Given these protonation state assignments and assuming Mn(iii)Mn(iv)_3_ oxidation states for the OEC, an S_2_ state model with both W1 and W2 ligands to Mn4 assigned as H_2_O includes 2065 atoms and has a total charge of zero. Consequently, the deprotonation of water molecules, such as in models where W2 is OH, results in anionic models. Complete coordinates are provided in PDB format as ESI.†

### 2.2. QM regions for multilayer calculations

To combine the accuracy of QM approaches with the explicit treatment of the OEC environment in this large model, the calculations were performed using a multilevel approach combining two QM levels of theory (QM1/QM2) *via* the 2-layered ONIOM method^127^ with H link-atoms using electrostatic embedding, as implemented in Orca 5.0.3.^128^ The high-level region (QM1) is treated at the DFT level, while the lower-level region (QM2) is treated with the semiempirical Extended Tight-Binding GFN2-xTB method.^123,129^ The latter has been shown to reproduce crystallographic structures of metalloenzymes with high accuracy.^129^ This choice of a semiempirical QM method for the low-level layer is of course considerably more expensive than molecular mechanics methods typically employed in QM/MM calculations, however modern implementations enable its use for systems of several thousand atoms. Crucially, the gain in accuracy and reliability is considerable because in this way we do not rely at all on fixed force-field parameters, we have the ability of fully adapting the environment to changes occurring in the QM1 region, to optimize the QM2 region simultaneously and in response to changes in the QM1 region, both the QM2 region internally and the QM1/QM2 embedding do not simply incorporate electrostatic interactions but also full polarization at a QM level. Given that the extent of the model from the OEC core is close to the Coulomb cutoff distance typically employed in QM/MM calculations, the present DFT/xTB construction can be considered as superior to any QM/MM model of the OEC previously used by us or other groups.

**Fig. 4:**
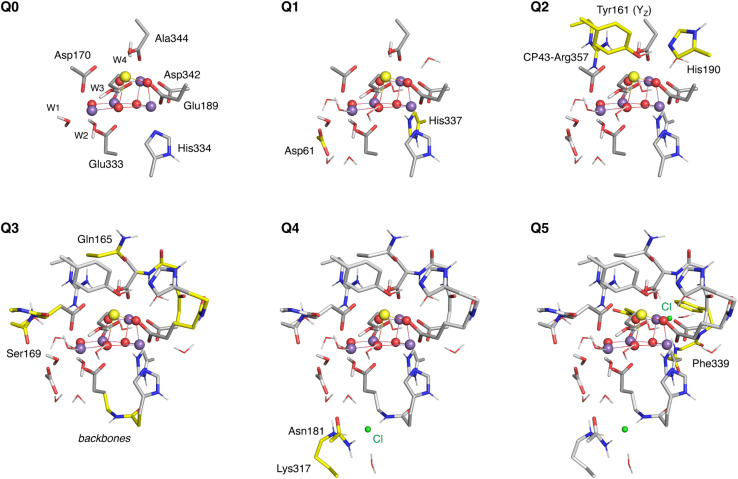
The six QM regions defined in this study, where colored residues indicate the additions made at each enlargement step. Hydrogen atoms are omitted for clarity.

Six QM1 regions of increasing size (Q0–Q5, Fig. 4) were defined in order to examine the convergence of structural and magnetic properties of the OEC with respect to the size of the QM1 region. The smallest region, Q0, contains 81 atoms and includes the Mn_4_CaO_5_ cluster and water ligands W1–W4 as well as the side chains of the first-sphere ligands His332, Glu189, Asp342, Glu354, Ala344, Asp170, and Glu333. The larger Q1 region (120 atoms) additionally includes residues Asp61 and His337, as well as 7 crystallographic water molecules that form hydrogen bonds with O4 (HOH505), Asp61 (HOH552), W2 (HOH554), W3 (HOH615, HOH510, and HOH590 connected with them), and O1 (HOH535). Q2 (164 atoms) expands by including Tyr16, its hydrogen bonding partner His190, and Arg357. In Q3 (229 atoms) the backbones of residues His332-Glu333, Glu189-His190, Asp342-Leu343- Ala344, and Ser169-Asp170-Gly171 are added, as well as the side chain of Ser169 and of Gln165 that form hydrogen bonds with HOH505 and W4, respectively. Q4 and Q5 successively include the proximal and distal chloride ions and their immediate environment in the DFT region. Q4 (257 atoms) includes Cl402 and its hydrogen bonding Lys317, Asn181, and a crystallographic water molecule HOH560. Q5 (301 atoms) additionally contains the second chloride ion Cl403 and HOH519, HOH697, Phe339, and the Asn338 and Gly353 backbones, all of which interact with the chloride. Convergence of the QM1/QM2 protocol was studied using models of the low-spin S_2_ state with all oxo-bridges modeled as deprotonated and all water ligands W1–W4 modeled in the H_2_O form.

### 2.3. Geometry optimizations

Starting from the crystallographic coordinates, the missing hydrogen atoms were added automatically using the Mercury software,^130^ and subsequently, the hydrogen bonding network was manually constructed. Initially, only the positions of the hydrogen atoms were optimized with GFN2-xTB, keeping heavy atoms constrained in their crystallographic positions. The next step was the optimization of the positions of all crystallographic water molecules using GFN2-xTB, while keeping all residues and the OEC constrained. The resulting structures with GFN2-xTB optimized H-atoms and H_2_O molecules, were used as starting coordinates for all geometry optimizations of the S_2_ state variants. Initially, geometry optimizations were performed with using the r^2^SCAN^131^ functional for the QM1 region and the structures were subsequently refined with the hybrid functional B3LYP.^132,133^ All geometry optimizations were carried out including D4 dispersion corrections,^134^ assuming a total high-spin state (*i.e.* ferromagnetic alignment of all Mn ions, multiplicity 14). In all calculations, tight convergence criteria were applied (TightSCF in Orca convention), and scalar relativistic effects were included using the zeroth-order regular approximation (ZORA)^135–138^ Hamiltonian. The ZORA-recontracted def2-TZVP(-f) basis sets were used for all atoms, except for C and H, for which the smaller ZORA-def2-SVP basis sets were used.^139^ In order to increase the speed of the calculations, the resolution of identity (RI) approximation^140^ and the chain of spheres (COSX)^141^ approximation to the Hartree–Fock exchange term were employed combined with the decontracted auxiliary SARC/J Coulomb fitting basis sets.

During geometry optimizations, all atoms of the QM1 region as well as atoms of the QM2 region within a radius of ∼10 Å around the Mn_4_CaO_5_ cluster, were left to fully relax (605 atoms in total, Fig. 3), whereas atoms in the outer layer of the QM2 region (1460 atoms) were constrained in their crystallographic positions – following the preliminary hydrogen-only and H_2_O optimization – to maintain the secondary structure of the protein matrix.

### 2.4. Calculations of magnetic and spectroscopic properties

Calculations of magnetic and spectroscopic properties were carried out on the QM1 region without including the QM2 region. The broken symmetry-DFT (BS-DFT) approach was used, employing the hybrid *meta*-GGA TPSSh functional,^142^ which has been previously used successfully for magnetic property calculations.^30,99,106,112,143–149^ Pairwise Mn–Mn exchange coupling constants, *J*_*ij*_, were computed by singular value decomposition to solve an overdetermined system of equations based on the Ising Hamiltonian, involving the energies of the high spin as well as of all BS determinants of the system. Subsequently, the isotropic Heisenberg–Dirac–van Vleck (HDvV) Hamiltonian:
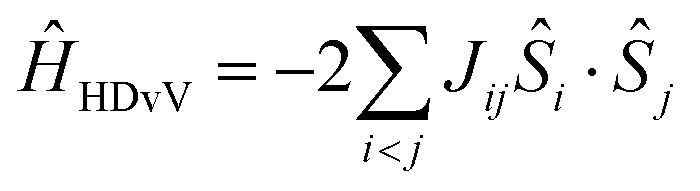
was diagonalized using the *orca_eca* utility to obtain the full set of spin eigenstates. Moreover, based on the spin projection coefficients of each Mn center obtained from the *orca_eca* utility, we selected the BS determinant that best represents the ground state electronic structure for each model. The selected determinants were later used for the calculations of hyperfine coupling tensors and XAS spectra.

For the calculation of ^55^Mn and ^14^N hyperfine coupling tensors the ZORA-def2-TZVP basis sets of Mn and N were modified with fully decontracted s-functions with three additional steep primitives with exponents 2.5, 6.25, and 15.625 added to the core,^150^ and locally dense radial grids were used (integration accuracy of 11 for Mn and 9 for N in ORCA convention). The spin–orbit coupling operator was calculated using the complete mean-field approach and “picture change” effects originating from the use of the scalar relativistic Hamiltonian were also included. The computed hyperfine coupling constants (denoted “raw” values) were subsequently transformed into on-site hyperfine coupling constants by applying previously reported spin projection techniques so that they could be meaningfully compared to experimental values. These methods have been previously benchmarked on dinuclear Mn complexes.^106,149,151^

The XAS spectra were calculated using the TD-DFT method with the TPSSh functional and the Tamm–Dancoff approximation.^152^ A shift of +36.3 eV was applied to the excitation energies to correct for systematic methodological deviations, as established in previous benchmarking studies on Mn systems.^153,154^ The XAS spectra of each Mn ion were computed with 150 roots. The spectra for each model were generated by summing the spectra of all four Mn ions and applying a Lorentzian peak broadening of 1.1 eV.

## 3. Results

### 3.1. Convergence of DFT/xTB geometries

Our first goal is to ensure a converged ratio between QM1/QM2 layer sizes so that geometric and electronic/spectroscopic parameters are converged. To this end, we use a progression of six QM1 (DFT) regions of increasing size (Q0–Q5, Fig. 4), as described in the Computational details. For the purposes of this investigation we used the open cubane model that is generally accepted to represent the low-spin form of the S_2_ state. Starting from the crystallographic coordinates of heavy atoms, r^2^SCAN/GFN2-xTB geometry optimizations were performed in two steps. First, only the QM1 region was optimized while keeping all atoms of the QM2 region constrained, and subsequently, both the QM1 and the structurally active part of the QM2 region were simultaneously optimized (605 atoms in total, see Fig. 3 and Computational details). Selected interatomic distances of all structures are given in Tables S1 and S2.† Before proceeding with the presentation of the results, we note that all optimized models have valence distribution [Mn1, Mn2, Mn3, Mn4] = [iii, iv, iv, iv], as indicated by the calculated Mn spin populations (Tables S3 and S4†). In the following, we examine how geometric parameters of the OEC, exchange coupling constants, and hyperfine coupling constants converge with respect to the size of the QM1 region.

Focusing first on structural parameters (Table 1), we observe that convergence of the geometry of the cluster with respect to the size of the QM1 region is very quick, and Q3 (229 atoms) is practically identical to the larger Q4 and Q5 regions. The most important differences between model Q0 and the converged geometry are in Mn1–Mn4 and Mn2,3,4–Ca distances and are up to ∼0.1 Å. In models Q1 and Q2, the Mn1–Mn4, Mn1–O5, and Mn4–O5 distances are ∼0.06 Å shorter than in the converged models. Compared to Q2, model Q3 contains additionally the backbones of first-sphere amino acids. Thus, all residues that form hydrogen bonds with the cluster, in addition to the peptide bonds of the first-sphere amino acids need to be included in the DFT-optimized region to ensure converged geometries. Crucially, further expansion of the QM1 region to include either only the proximal (in Q4) or both the proximal and the distal chloride anions (in Q5) along with the groups that form hydrogen bonds with them, has negligible effect on the optimized structural parameters. Overall, full convergence with respect to structural parameters is achieved in model Q3.

**Table 1:** Selected key interatomic distances (Å) for the geometry-optimized models Q0–Q5

	Mn1–O5	Mn4–O5	Mn1–Mn2	Mn2–Mn3	Mn3–Mn4	Mn1–Mn3	Mn1–Mn4	Mn1,2,3,4–Ca
Q0	3.92	2.93	2.72	2.73	2.70	3.21	4.72	3.48, 3.29, 3.54, 3.92
Q1	3.88	2.90	2.72	2.72	2.70	3.20	4.71	3.51, 3.35, 3.56, 3.88
Q2	3.91	2.90	2.73	2.72	2.70	3.20	4.70	3.48, 3.39, 3.62, 3.91
Q3	3.87	2.96	2.73	2.72	2.70	3.22	4.77	3.46, 3.38, 3.58, 3.87
Q4	3.86	2.96	2.73	2.72	2.70	3.22	4.77	3.46, 3.39, 3.58, 3.86
Q5	3.87	2.95	2.73	2.72	2.70	3.22	4.76	3.46, 3.39, 3.59, 3.87

Turning our attention to calculated Mn–Mn exchange coupling constants (Table 2), we observe that all models have the same exchange coupling pattern, with antiferromagnetic coupling between Mn1–Mn2 and Mn3–Mn4, and ferromagnetic coupling between Mn2–Mn3, leading to a *S* = 1/2 ground state, as known from past studies, and the first magnetically excited state has *S* = 3/2. The exchange coupling constants converge quickly, already from Q2. The largest difference between Q0 and the converged models is observed for *J*_34_, which in Q0 exceeds the converged value by ∼8 cm^−1^, leading to ∼10 cm^−1^ higher energetic separation between the ground and the first excited state. The energetic separation from the ground state is converged in model Q2 at 23 cm^−1^, consistent with the EPR-determined value of ∼30 cm^−1^.^41^

**Table 2:** Computed exchange coupling constants *J*_*ij*_ (cm^−1^) and energy differences (cm^−1^) between the ground (*S* = 1/2) and the first magnetically excited state (*S* = 3/2) of the geometry-optimized models Q0–Q5

	*J*_12_	*J*_13_	*J*_14_	*J*_23_	*J*_24_	*J*_34_	Δ*E*_ES–GS_
Q0	−25	2	13	19	4	−20	34.1
Q1	−20	2	11	22	3	−14	25.0
Q2	−20	2	12	18	2	−13	23.2
Q3	−21	1	10	17	2	−12	23.2
Q4	−21	1	10	16	2	−12	23.5
Q5	−21	2	9	16	2	−12	22.1

Finally, we examine the convergence of Mn hyperfine coupling constants as reporters of the local electronic structure of the Mn ions (Table 3). We note that these are the “raw” calculated values using the lowest-energy BS determinant (*αββα*), which means that they are unscaled and unprojected and cannot be directly compared to experimental values. The hyperfine coupling constants are more sensitive to the size of the DFT region than the geometries and exchange coupling constants and are considered converged at the QM1 size of Q3.

**Table 3:** Calculated values for the unprojected ^55^Mn isotropic hyperfine coupling constants (MHz) of the geometry-optimized models Q0–Q5

	Mn1	Mn2	Mn3	Mn4
Q0	−437	406	424	−463
Q1	−442	413	420	−477
Q2	−448	413	421	−477
Q3	−444	410	417	−477
Q4	−442	411	417	−476
Q5	−440	411	417	−474

**Fig. 5:**
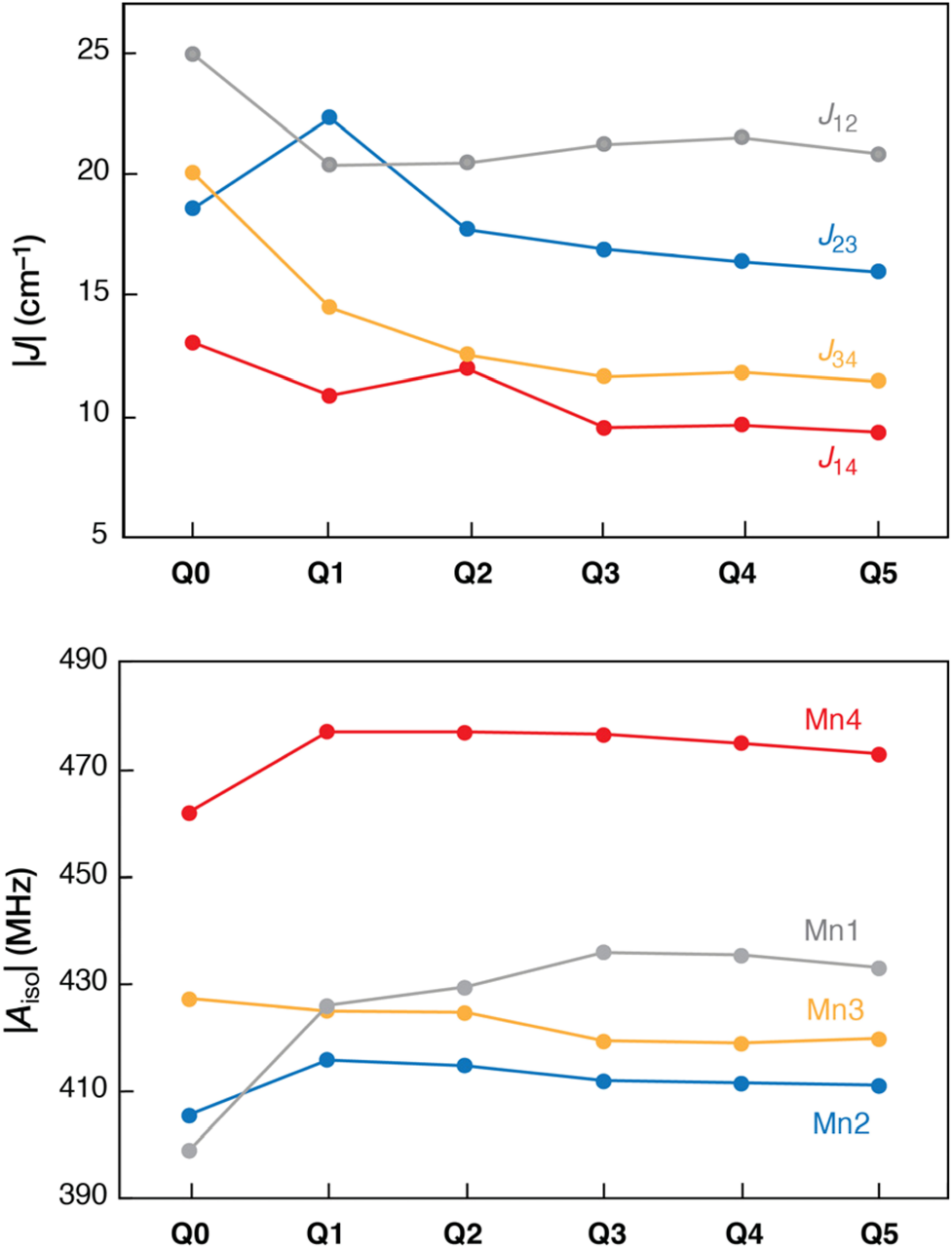
Convergence of the Mn pairwise exchange coupling constants |*J*_*ij*_*|* (up), and of the isotropic Mn hyperfine coupling constants |*A*_iso_| (down) with increasing QM size for the DFT/xTB optimizations.

Overall, our results show that both the geometric and the more sensitive magnetic parameters converge at Q3 (Fig. 5). Notably, when all atoms in the QM2 region are kept constrained in their crystallographic positions, convergence occurs slower but still at Q3 (Tables S1–S8†). Further increasing the DFT-optimized region in the DFT/xTB model to include the proximal and distant chloride ions does not impact the calculated properties. At the same time, our results show that small QM regions are unreliable for simulating spectroscopic parameters.

We are confident that this conclusion applies to more approximate two-layer approaches such as QM/MM. The size and nature of the QM region needed to achieve convergence in QM/MM calculations are inherently system-specific^155–160^ and may vary depending on the properties of interest. This means that geometry, reaction barriers, and spectroscopic parameters may each converge at different QM region sizes. Specifically for the OEC, several different QM/MM models have been developed with different QM sizes ranging from ∼100 to ∼400 atoms.^36,121,161,162^ The convergence of the QM region for both geometric and spectroscopic properties has been systematically investigated by Retegan *et al.*,^156^ who concluded that all residues and water molecules hydrogen-bonded to the inorganic core should be included in the QM region (this corresponds to size Q2 in this work). Therefore, the QM/xTB and QM/MM models of the OEC have similar convergence behavior regarding the DFT (QM1) region.

These results encourage the use of QM/xTB not only for the study of the OEC but also for other metalloenzymes, considering its notable practical advantages over conventional QM/MM. These include flexible charge response and polarization of the environment at a QM level and a “black-box” implementation that eliminates the need for force field parameterization, which is problematic in cases where chemically uncommon groups with complex electronic structures are present. Having established a converged QM/xTB approach, we proceed with Q4 in which the QM1 size is beyond the point of convergence, in order to ensure maximum flexibility that will enable accommodation of significant structural distortions on the high-spin S_2_ models, especially those that involve extended deformations from the crystallographic models.

### 3.2. Models of the high-spin S_2_ state

**Fig. 6:**
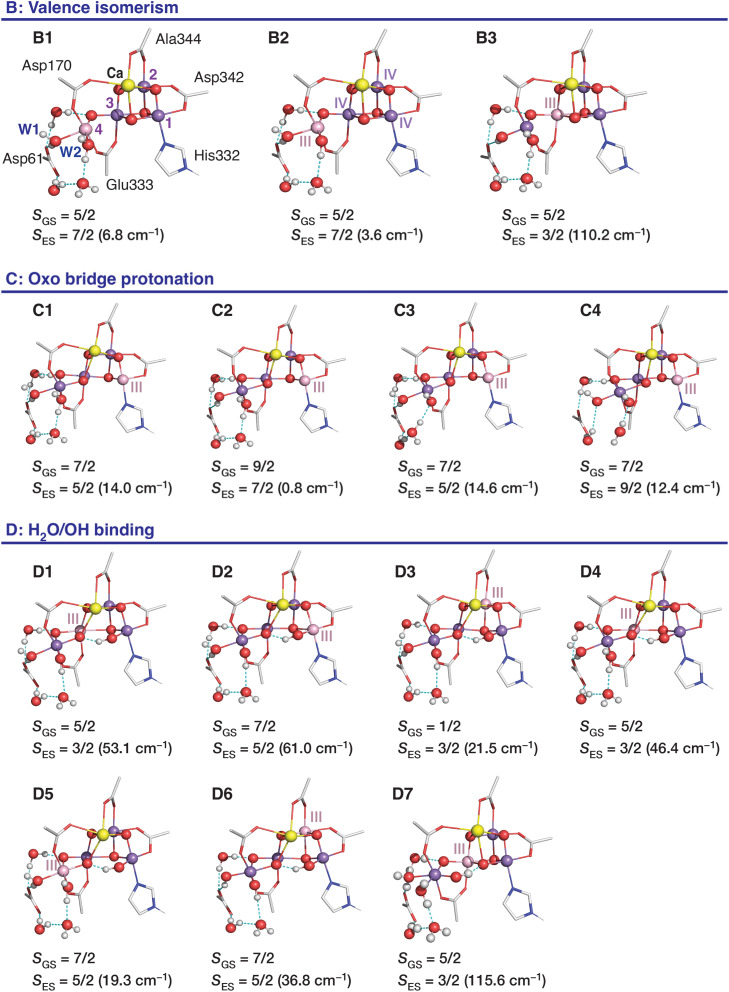
Representations of the core structures of the S_2_ high-spin variations. Mn(iv) ions are represented in purple color, Mn(iii) in light pink, Ca in yellow, N in blue, and O in red. Only a limited part of the total computational models is shown for a better depiction of the structural differences.

Computational high-spin models of the S_2_ state have been presented before in independent studies and have been used as a basis for interpreting spectroscopic and kinetic observations, as well as to suggest possible S_2_ → S_3_ transition pathways.^99,101,102,113,116,118–120,162^ Herein, we constructed and optimized models that describe all literature suggestions using the current computational setup that represents the most elaborate QM-based approach used for the OEC. The inorganic cores of all high-spin S_2_ state models are shown in Fig. 6 and S1,† key structural parameters are given in Table 4, and Mulliken spin populations on the Mn ions in Table S9.† Since the protonation states of terminal water ligands cannot be definitively known^163–167^ and considering that they may vary under different experimental conditions, we examine models with varying protonation states of W1 and W2. The high-spin candidate models are divided into three groups, labeled B, C, and D, with each model further distinguished by a number indicating the specific conformational variant. In the following, the structural, spectroscopic, and energetic parameters of the high-spin models will be compared to those of the low-spin open cubane models A1 and A2, with W2 = H_2_O and OH, respectively.

**Table 4:** Selected interatomic distances (Å) for all high-spin variations

	Mn1–Mn2	Mn2–Mn3	Mn3–Mn4	Mn1–Mn3	Mn1–O5	Mn4–O5
A1	2.740	2.730	2.706	3.241	2.977	1.831
A2	2.724	2.740	2.734	3.249	2.891	1.862
B1	2.717	2.725	3.058	2.853	1.839	3.012
B2	2.713	2.743	3.062	2.859	1.842	3.016
B3	2.727	2.751	3.101	2.909	1.771	3.185
C1	2.757	2.721	2.820	3.258	3.036	1.834
C2	2.752	2.724	2.845	3.193	2.888	2.009
C3	2.750	2.726	2.831	3.244	2.978	1.861
C4	2.754	2.718	2.919	3.058	2.457	2.151
D1	2.735	2.922	2.785	3.650	3.544	1.811
D2	2.887	2.758	2.777	3.597	3.440	1.794
D3	2.815	2.867	2.774	3.423	3.412	1.794
D4	2.755	2.842	2.782	3.528	3.495	1.793
D5	2.763	2.822	2.765	3.522	3.407	1.836
D6	2.824	2.895	2.824	3.431	3.406	1.906
D7	2.715	2.761	3.231	2.962	1.782	3.470

Models in group B align with the original valence isomerism hypothesis,^99^ which states that the position of the unique Mn(iii) ion of the cluster changes position in the high-spin S_2_ state. This leads to shortening of the Mn1–O5 bond, forming the closed cubane conformation of the cluster. Models B1 and B2 feature a [iv, iv, iv, iii] Mn oxidation state distribution (Table S9†). They have been constructed with W1 = H_2_O and with W2 = H_2_O and OH, respectively. In both models the pseudo Jahn–Teller (JT) elongation axis of Mn4(iii) aligns with the W1–Mn4–O5 direction (JT axes of all models are shown in Fig. S1†).

Geometry optimization starting from the coordinates of B1 and deprotonating W1, led to model B3 with Mn oxidation state distribution [iv, iv, iii, iv] and a five-coordinate Mn4(iv) ion. B3 is reported for the first time as an alternative closed cubane valence isomer of the S_2_ state. A recent report suggested a closed cubane model with W1 = OH which seems to have [iv, iv, iv, iii] Mn oxidation states.^168^ We note that no stable structure with W1 = OH and [iv, iv, iv, iii] Mn oxidation states could be located in our geometry optimizations, in line with a previous study.^166^ This could be attributed to the fact that deprotonation of W1 would make it more nucleophilic and positioning a more nucleophilic ligand along the occupied d_*z*^2^_ orbital of Mn(iii) is expected to be energetically unfavorable. Alternatively, the orientation of the d_*z*^2^_ orbital of Mn(iii) could change, as suggested in ref. 168, but this is also expected to be unfavorable given the long Mn4–O5 distance. Indeed, proton transfer from W1 to Asp61 in the closed cubane form with [iv, iv, iv, iii] Mn oxidation state distribution in the presence of the oxidized tyrosyl radical leads to fast electron transfer from Mn4 to 
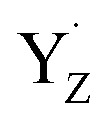
.^118,169^ Thus, deprotonation of W1 facilitates Mn4(iii) oxidation. In our models, which incorporate neutral Y_Z_, deprotonation of W1 shifts the position of the unique Mn(iii) in the Mn3 position.

Type C models feature a protonated O4 oxo bridge, according to the hypothesis by O'Malley and coworkers that proton isomers may rationalize the high- and low-spin forms of the S_2_ state.^102^ All type C models have an open cubane conformation and a [iii, iv, iv, iv] Mn oxidation state distribution. In C1 both W1 and W2 are H_2_O, in C2 W1 = OH and W2 = H_2_O, in C3 W1 = H_2_O and W2 = OH, and in C4 both W1 and W2 are OH. Protonation of the O4 oxo bridge leads to a distance increase of ∼0.1 Å for the OH-bridged Mn3–Mn4 relative to the low-spin models, while all other Mn–Mn distances remain essentially the same. Our expanded DFT/xTB models enable us to investigate how O4 protonation may alter the hydrogen bonding network of the O4 water channel (Fig. S2†). Upon O4 protonation, the directions of hydrogen bonds in the O4 channel up to ∼8 Å away from the cluster are expected to change in order to accommodate the additional proton on O4.

Type D models include an additional hydroxyl ligand coordinated to the cluster, in line with the early substrate water binding hypothesis proposed by Pushkar and coworkers.^101^ Models D1–D6 feature a hydroxyl group as a sixth ligand on Mn1. When the protonation states of the terminal water ligands were modeled as W1 = H_2_O and W2 = OH, three valence isomers D1, D2, and D3, were located, with Mn(iii) ion at the Mn3, Mn1, and Mn2 position, respectively. Models D1 and D3 imitate the corresponding structures of the previous work (therein named A and C).^101^ Model D2 was proposed by Siegbahn as an intermediate during the S_2_ → S_3_ transition,^113^ but the [iv, iv, iv, iii] Mn oxidation state distribution was found energetically favorable over [iii, iv, iv, iv]. By contrast, our calculations showed that in models of type D, both terminal ligands on Mn4 need to be doubly protonated to stabilize the Mn4(iii) oxidation state. With both W1 and W2 ligands in the H_2_O form, Mn(iii) can be in the Mn3 or Mn4 position, represented by the valence isomers D4 and D5, respectively. Notably, the Mn(iii) ions of all optimized S_2_ models are axially elongated (elongation axes are denoted in Fig. S1†), due to JT distortion stabilizing the singly occupied d_*z*^2^_ orbital of Mn(iii) ions. The only exception is Mn4(iii) in D5, which is slightly axially compressed along the W1–Mn4–O5 axis. In D6, W1 and W2 were modeled as OH, and Mn(iii) occupies the Mn2 position. Finally, an alternative early water binding scenario was examined involving the closed cubane conformation.^114,115^ Model D7 arises from model B1 with coordination of an additional hydroxyl as a sixth ligand to Mn4, *i.e.* it is the closed cubane isomer of D5. In D7, Mn(iii) is at the Mn3 position.

At this point, it is worth examining whether structural parameters can be used to distinguish between the various high-spin forms of the S_2_ state. For the low-spin S_2_ state, structural characterization has been performed using EXAFS^170–172^ and crystallography,^124,173–176^ both of which are consistent with three short (∼2.7 Å) and one long (∼3.2 Å) Mn–Mn distances in the Mn_4_CaO_5_ cluster, in agreement with quantum chemical models for the low-spin form (Table 4). In contrast, fewer structural data exist for the high-spin forms of the S_2_ state. To date, only the high-spin form associated with the *g* ≈ 4.1 signal has been structurally characterized by EXAFS,^101,103,104^ with no crystallographic data available for any high-spin form. However, EXAFS results for the *g* ≈ 4.1 are contradicting. Early EXAFS studies by Liang *et al.*,^103^ which were later confirmed by Pushkar *et al.*,^101^ suggest an elongation of one short Mn–Mn distance by ∼0.1 Å compared to the low-spin form, yielding two short (∼2.7 Å) and one elongated (∼2.85 Å) Mn–Mn distances. The attribution of features at *ca.* 3.2–3.3 Å and beyond remains inconclusive in both studies. These contrast with another EXAFS study by Chatterjee *et al.*^104^ who reported a different Mn–Mn arrangement for the *g* ≈ 4.1 form, with two short (∼2.7 Å) and two long (∼3.3 Å) Mn–Mn distances. The diverging conclusions were attributed to differences in the data quality, experimental conditions, and settings.^101,104^

The Mn–Mn distances of the high-spin models are given in Table 4. All models have three short distances, in line with Liang *et al.*,^103^ and Pushkar *et al.*^101^ None of the possible models considered is consistent with the interpretation of Chatterjee *et al.*^104^ Differentiating between models B and C using EXAFS would be particularly challenging. In models of group C, protonation of the oxo-bridge O4 elongates the Mn3–Mn4 distance by ∼0.1 Å, which has also been observed in synthetic Mn(iv)–Mn(iv) oxo complexes.^110^ Meanwhile, the long Mn1–Mn3 distance remains similar to that of the low-spin form. Closed cubane models of type B and model D7 feature shorter Mn1–Mn3 distances relative to open cubane structures, around ∼2.9 Å, and elongated Mn3–Mn4 distances, higher than 3 Å. The distinctive structural feature of models D1–D6 is the elongated “long” Mn1–Mn3 distance from 3.2 Å in the low-spin form to longer than 3.4 Å after the coordination of a hydroxyl group on Mn1. Consequently, the proposed early water-binding scenario in the open cubane S_2_ state could be tested in the future based on the long Mn–Mn distances. Since it is still impossible to distinguish between the proposed high-spin models from available structural data, spectroscopic and energetic criteria are essential and will be discussed in the following.

### 3.3. Magnetic properties

#### 3.3.1. Exchange couplings and spin states

The main feature of these structural models that can make them potentially relevant for the discussion of the spectroscopy of the S_2_ state is their total spin: all three types of model have been suggested to have a sufficiently high ground-state spin to render them consistent with low-field EPR signals observed in the S_2_ state of the OEC. Given that the computational studies performed in the past employed diverging structural models and theoretical approaches, here we have the opportunity to revisit this question using a common and state-of-the-art computational setup. The calculated exchange coupling constants for all models are given in Table 5, along with the spin of the ground and the first excited states, and the energy difference between them.

**Table 5:** Calculated exchange coupling constants *J*_*ij*_ (cm^−1^), ground spin state (*S*_GS_), first excited spin state (*S*_ES_), and their energy separation Δ*E*_ES–GS_ (cm^−1^) for all models of the S_2_ state

	*J*_12_	*J*_13_	*J*_14_	*J*_23_	*J*_24_	*J*_34_	*S*_GS_	*S*_ES_	Δ*E*_ES–GS_
A1	−21	2	8	16	2	−12	1/2	3/2	23.5
A2	−27	4	8	13	2	−12	1/2	3/2	24.1
B1	29	7	6	30	2	−17	5/2	7/2	6.8
B2	27	11	9	27	1	−22	5/2	7/2	3.6
B3	34	−8	2	−57	0	−30	5/2	3/2	110.2
C1	−16	3	9	16	0	9	7/2	5/2	14.0
C2	−19	13	4	22	0	9	9/2	7/2	0.8
C3	−19	6	7	16	0	5	7/2	5/2	14.6
C4	−28	17	4	26	0	9	7/2	9/2	12.4
D1	19	−39	−3	14	6	−51	5/2	3/2	53.1
D2	26	−31	0	8	1	−26	7/2	5/2	61.0
D3	−19	18	6	8	−4	−18	1/2	3/2	21.5
D4	18	−36	−5	17	7	−71	5/2	3/2	46.4
D5	9	−9	2	4	0	−143	7/2	5/2	19.3
D6	6	1	1	17	−1	−48	7/2	5/2	36.8
D7	30	−21	3	−67	−10	−35	5/2	3/2	115.6

First, we observe that all of the models indeed display high-spin ground states with the exception of D3, which has a low-spin ground state with *S* = 1/2. The ground states computed for all other models are either 5/2 or 7/2, which are the two spin states conventionally associated with the signals *g* ≈ 4.1 (ref. 177 and 178) and *g* ≈ 5.^96^ In the case of C2 the ground state is predicted to be *S* = 9/2, but this is nearly degenerate with an *S* = 7/2 state.

All models of type B have *S* = 5/2 ground states. In the low-spin type A models, Mn1–Mn2 are strongly antiferromagnetically coupled, Mn2–Mn3 are weakly ferromagnetically coupled, and Mn3–Mn4 are weakly antiferromagnetically coupled. The remaining Mn–Mn exchange couplings (*J*_13_, *J*_14_, *J*_24_) are smaller. The distinctive feature of type B models compared to the low-spin models is the ferromagnetic coupling between Mn1 and Mn2. In B1 and B2, the Mn(iv)_3_Ca cubane is a high-spin *S* = 9/2 unit coupled antiferromagnetically to the Mn4(iii) ion. The strength of this antiferromagnetic coupling thus determines the ground state spin of the cluster.^144^ As shown in Section 3.1, the *J*_34_ coupling is the most sensitive to the size of the DFT (QM1) region. Notably, calculations by Krewald *et al.*^144^ on a smaller cluster model corresponding to B1 predicted a significantly weaker *J*_34_ of −2.6 cm^−1^ resulting in a *S* = 13/2 ground state, while for B2 a *J*_34_ of −7.3 cm^−1^ was predicted giving the *S* = 5/2 and *S* = 7/2 lowest energy states nearly degenerate. Herein, using the largest models reported, we find *J*_34_ in the order of *ca.* −20 cm^−1^ for B1 and B2, with the *S* = 7/2 first excited state being more than 3 cm^−1^ higher than the ground state. The exchange coupling topology of B3, in which Mn(iii) is in the Mn3 position, differs from the other closed cubane models because Mn3 is strongly antiferromagnetically coupled to Mn1 and Mn2. Therefore, the lowest energy BS determinant has the *ααβα* spin configuration, in contrast to the *αααβ* predicted for B1 and B2. Moreover, the ground state is energetically well separated from the first magnetically excited state by more than 110 cm^−1^. The experimentally determined energy differences between the two lowest states of the spin ladder are in the order of ∼30 cm^−1^ for the *g* ≈ 4.1 EPR signal,^178^ therefore among all *S* = 5/2 models B3 deviates most from this value.

As already discussed by O'Malley and coworkers,^102^ protonation of the O4 bridge attenuates the superexchange interaction between Mn3 and Mn4, to the point that they become ferromagnetically coupled, leading to a high-spin ground state. Protonation of the oxo bridges is known to switch off the dominant antiferromagnetic exchange pathways of Mn–oxo cores.^110,111,179^ All type C models have a *S* = 7/2 ground state (in C2 nearly degenerate with *S* = 9/2), whereas the *S* = 5/2 excited state is higher by at least 14 cm^−1^. Notably, C3 and C4 correspond to the models proposed to have *S* = 5/2 and *S* = 7/2 ground states, respectively,^180,181^ using smaller cluster models.

Group D is the only subset of high-spin forms that contains isomers with both *S* = 5/2 and *S* = 7/2 ground states. Among these models, D1 and D3 resemble those originally reported by Pushkar *et al.*^101^ Contrary to those results, we find that D3 has a low-spin *S* = 1/2 ground state. In all high-spin type D models, Mn3 and Mn4 are strongly antiferromagnetically coupled. At this point, it is worth noting that while in the original work by Pushkar *et al.*^101^ His337 was modeled in the neutral form, motivated by the fact that the high-spin signal is more intense at high pH, here His337 was considered as doubly protonated in all models. However, we confirmed that the protonation state of His337 does not affect the ground spin states of the models, with the only one exception being the quasi-degenerate model B2, which has a *S* = 7/2 ground state when His337 is assumed as neutral (Table S11†). Models D1 and D4, which differ only in a terminal ligand (W2) protonation state, both have [iv, iv, iii, iv] Mn oxidation state distribution and similar exchange coupling topologies, leading to *S* = 5/2 ground states. Model D7, with the same valence distribution but a closed cubane conformation, also has an *S* = 5/2 ground state. It arises from a similar coupling scheme to B3, featuring a distinctively large antiferromagnetic coupling between Mn2 and Mn3, which leads to very large energetic separations from the first magnetically excited states with *S* = 3/2. The remaining high-spin D type models, D2, D5 and D6, all have *S* = 7/2 ground states, despite having different valence distributions and protonation states.

Overall, all examined models except D3 are in principle “capable” of yielding a spin state with *S* = 5/2 or 7/2. This, taken in isolation, makes them plausible candidates for one or more of the high-spin signals of the S_2_ state, but this criterion is not sufficient to differentiate between the models.

#### 3.3.2. Plausible connections to EPR signals

Since the effective *g* values and shapes of EPR signals of *S* > 1/2 species may often be reproduced using more than one alternative combinations for the total spin and zero-field splitting parameters of the system, there is not in general a unique correspondence between total spin (*S*) and effective *g*. From the point of view of quantum chemistry, the correlation of the computational data to experimental data has been exclusively based on the ground spin state by making reasonable assumptions about all other parameters in the spin Hamiltonian. Ideally, both the calculated zero-field splitting tensor and all pairwise Mn–Mn exchange coupling constants for a given structure would be used to construct the spin Hamiltonian. However, there are important limitations, including the fact that calculations of the total zero-field splitting in multi-spin systems are extremely challenging for computational chemistry and necessitate the use of aggressive approximations, and second, because simplifying assumptions regarding the strong-exchange limit and the appropriate treatment of effective Hamiltonians are not necessarily valid. Therefore, quantum chemical calculations cannot directly provide effective *g* values of the cluster; they can only provide selected individual spin Hamiltonian parameters that may be used as partial starting points for EPR simulations.

Experimentally, the *S* can be assigned with confidence using multifrequency EPR experiments. These have only been reported for the *g* = 4.1 signal of spinach PSII, providing a definitive assignment to a *S* = 5/2 species, which allows determination of the corresponding zero-field splitting *D* and *E*/*D* values.^177,178^ For all other higher-*g* S_2_ signals, it is merely accepted that they correspond to *S* ≥ 5/2 species. Notably, the so-called *g* ∼ 5 signal generated by near-infrared illumination of the S_3_ state,^95^ which resembles the *g* = 4.75 signal of the S_2_ in high pH,^19^ has been assigned to a *S* = 7/2 species.^96^ In that case, the total spin is constrained by the isotropic form of the signal, which can only arise in the region of the observed *g* between 4.6 and 4.8 from the transition between the m_*S*_ +3/2 and −3/2 sublevels of a *S* = 7/2 system. This also restricts the zero-field splitting *D* and *E*/*D* values.^96^ Even though these conclusions could reasonably be applied to the *g* = 4.75 signal of the S_2_ state, multifrequency EPR experiments would still be useful to provide definitive assignments. An additional key question that future EPR experiments would address is whether the *g* = 4.75 signal of the S_2_ state^19^ and the *g* ∼ 5 signal of the near-infrared illuminated S_3_ state correspond to the same species. This could provide direct insight into the extensively debated S_2_ → S_3_ state transition.^32^ Regarding the higher-field signals (*g* > 5), it is difficult to even constrain the ground spin state possibilities based on the *g* values, thus multifrequency EPR experiments are essential. Overall, it should be appreciated that the available experimental and computational data do not allow a unique assignment of structural models to observed signals.

Another question is whether all observed higher-*g* EPR signals of the S_2_ state can be assigned to a specific type of model with different protonation states of the terminal water ligands and/or under minor structural perturbations. Alternatively, interconversion between different types of model would take place under different experimental conditions. The second case would testify to the pronounced structural plasticity of the cluster, suggesting that treatments that do not necessarily affect the first coordination sphere can nevertheless modify the conformation of the cluster. Our results enable us to discuss both scenarios.

Even though the ground spin state of models of types B and C is very sensitive to slight structural differences around Mn4 that affect *J*_34_ significantly, all models of type B have ground spin states *S* = 5/2 and all C models have *S* = 7/2 (or 9/2), which implies that neither open–closed cubane isomerism nor O4 protonation can easily explain both the *g* = 4.1 and the *g* = 4.75 signals. In fact, the *g* = 4.1 signal can be obtained either from models of type B or D, which have *S* = 5/2 ground spin states. Contrary to models of type B and C, interconversion between D type models can switch their spin state by valence isomerism or protonation state changes. Such interconversions are, for example, valence isomerism between D1 and D2, and between D4 and D5, deprotonation of D1 or D4 to D6, or open-to-closed cubane isomerism between D5 and D7.

Therefore, these results suggest that either all observed high-spin signals originate from type D models, in line with the early substrate binding hypothesis, or the cluster can sample high-spin conformations from more than one model type, possibly driven by externally imposed structural changes under different conditions or in different organisms.

Water exchange experiments indicated that a fast-exchanging substrate binds to the S_2_ state and is also bound in the S_3_ state, which means that both substrate water molecules are bound to the cluster already in the S_2_ state.^114,182,183^ If this extra ligand is a substrate, then B and D type models may coexist.^114^ Alternatively, water binding to Mn1 and deprotonation of the low-spin open-cubane, for example *via* a proton transfer mechanism proposed by Siegbahn,^113^ could generate D type structure(s). Overall, despite the fact that it remains hard to disqualify proposed models on the available magnetic data, we are able to constrain the possible scenarios about the origins of multiple high-spin signals to either only D type models or to a combination of models of different types. In the following, we examine what other parameters might help us to distinguish between the computational models.

### 3.4. Energetic considerations

Given that we practically cannot have a unique answer in terms of spin properties, we need to consider additional properties that can help us differentiate among the different possibilities. One such consideration is the relative energies, particularly in comparison to the low-spin state. This is not as straightforward as would be desirable because not all models are isomers. That is obviously the case for models of type D, which involve the coordination of an additional OH ligand compared to the low-spin form. Nevertheless, we can make such comparisons for specific subgroups of models, *e.g.* models of types B and C are compared to the low-spin form (A), while type D models are compared among themselves. Table 6 gives the relative DFT/xTB energies of the models within each of the four subgroups of isomers using the standard optimization setup (QM region of size “Q4”, *i.e.* with *ca.* 260 atoms) as well as single-point calculations with a drastically expanded QM region of *ca.* 600 atoms. This large QM region covers a radius of *ca.* 10 Å around the Mn_4_CaO_5_ cluster and is used to confirm convergence of the results and eliminate possible uncertainties arising from the xTB treatment of the protein matrix.

**Table 6:** Relative energies (in kcal mol^−1^) per group of isomers of the S_2_ variants, calculated with B3LYP/xTB using the “QM4” region of *ca.* 260 atoms defined in Section 3.1, as well as a QM1 region of *ca.* 600 atoms within a radius of ∼10 Å around the Mn_4_CaO_5_ cluster. The third column reports relative energies of the ∼260 QM regions in isolation, *i.e.* by removing the energetic terms deriving from the xTB layer

	DFT(∼260 atoms)/xTB	DFT(∼600 atoms)/xTB	DFT(∼260 atoms)
A1	0.0	0.0	0.0
B1	5.5	9.1	2.6
C2	32.4	33.7	21.6
C3	24.2	30.7	8.1
A2	0.0	0.0	0.0
B2	1.6	2.5	3.7
B3	2.6	8.9	23.6
C4	30.9	38.9	16.9
		38.87	
D2	0.0	0.0	0.0
D1	2.8	2.3	3.4
D3	3.3	4.0	5.4
D4	0.0	0.4	0.0
D5	1.5	0.0	0.7
D7	0.5	9.2	2.3

First, we observe that all high-spin candidates of types B and C are energetically higher than the low-spin form, which aligns with the expectation that the latter is predominant under native conditions. However, their relative energies compared to the low-spin form are drastically different. While type B models can be very close to the low-spin ground state, at energetic separations fully consistent with experiment^184^ (particularly models B2 and B1), all models of type C are strongly unfavorable in energetic terms. Specifically, O4-protonated models are all higher in energy by more than 30 kcal mol^−1^ in the case of the 600-atom QM region, while the lowest-energy C-type model with the standard 260-atom QM region (C3) is still more than 24 kcal mol^−1^ higher than the low-spin state. This is a crucial conclusion of the present work, because it means that C-type models are strictly inaccessible in energetic terms, therefore they cannot be observable. To ensure the reliable determination of the energetic behavior of the isomers in groups B and C, the relative energies were calculated using a variety of alternative approaches (Table S10†). Functionals with different Hartree–Fock exchange (HFX) percentages, *i.e.* B3LYP with default 20% HFX, B3LYP* (with 15% HFX), and TPSSh (with 10% HFX), all are mutually consistent and further support our conclusions. For greater accuracy, domain-based local pair natural orbital coupled-cluster with singles, doubles, and perturbative triples, DLPNO-CCSD(T),^185–188^ are employed here for the first time in a multilevel DLPNO-CCSD(T)/xTB framework. For these calculations the smaller QM region Q1 was used, defined in Section 2.2. The observed trend in energy differences between the LS and HS candidates remains consistent and confirms that on energetic grounds, C-type models are not plausible for high-spin signals of the S_2_ state.

Among valence isomers D1–D3, the most energetically favorable is D2, where Mn(iii) is on the Mn1 position. However, D1 is higher by a small margin of the order of 2 kcal mol^−1^, which suggests that in the hypothetical scenario of early substrate binding to Mn1, both valence isomers could be simultaneously present in the samples. The valence isomers D4 and D5 are also energetically close, while the closed-cubane isomer, D7, is considerably higher in energy, as revealed by the calculations using the enlarged QM region. Interestingly, the low-spin D3 isomer is the highest in energy and there are no other low-spin alternatives, which means that in the case of H_2_O/OH binding in the S_2_ state, the cluster could sample multiple high-spin conformations.

In some cases, the relative energies obtained for an isolated QM cluster equal to the QM region of ∼260 atoms and excluding the rest of the two-layer model are different than those obtained considering the whole 2065-atom region (Table 6, right column). Specifically, for models C2, C3, B3, and C4, the energy differences from the corresponding low-spin form may differ by more than 10 kcal mol^−1^ when a small QM region is considered in isolation. Given that the QM/xTB calculations that use the expanded ∼600-atom QM regions agree reasonably well with the standard QM/xTB calculations, we conclude that the GFN2-xTB treatment of the protein matrix provides a reliable estimate of the relative energies. This underscores the limitations of QM cluster modeling, as it cannot account for the (de)stabilizing effect of structural perturbations induced by alterations in the hydrogen-bonding network further from the Mn_4_CaO_5_ cluster. This also highlights that the QM/xTB multilevel approach is a powerful method that can achieve QM-level accuracy for unprecedentedly large systems, and thus we recommend its use in metalloenzymes instead of, or combined with, MM description of the rest of the protein matrix, for example, in QM/xTB/MM approaches.

The QM/xTB method employed here enables for the first time explicit incorporation of these intermediate-range effects at a quantum mechanical level and establishes with confidence that protonation of O4, and hence models of type C, are energetically implausible. The above methodological observations may also have implications for the study of the mechanism of the OEC. Many computational studies of crucial catalytic steps have been performed with restricted QM-only cluster models that ignore second-sphere energetic contributions even at the intermediate ranges covered in the present two-layer approach. It will be important to revisit such studies in view of the occasionally drastic alteration of energetics that can be obtained by consideration of a larger part of the environment at a quantum-mechanical level.

### 3.5. Hyperfine and superhyperfine coupling constants

The hyperfine and superhyperfine coupling constants of the high-spin signals may serve as independent criteria to evaluate the proposed models. Recent ESEEM experiments on spinach S_2_ state samples exhibiting the *g* = 4.1 signal, determined the ^14^N HFC at approximately 1 MHz.^178^ In prior computational studies, this has been used to support specific models that correspond to the present B2 and C3 models.^189^ Here, we calculated the HFCs of the His332 ^14^N coordinated to Mn1 (Fig. 6) for all twelve variations of the high-spin S_2_, excluding model D3 due to its low-spin ground state. The calculated isotropic ^14^N HFCs (*A*_iso_), given in Table 7, are within the narrow range of 0.5 to 0.9 MHz. We note that among the twelve high-spin variations, only models B1, B2, B3, D1, D4, and D7 that have a *S* = 5/2 ground state can be connected to the *g* = 4.1 signal. Their predicted ^14^N HFCs of these models range between 0.6 and 0.9 MHz and are consistent with experiment. Moreover, the determined nuclear quadrupole interaction (NQI) constant is around 2 MHz.^178^ The calculated NQI tensors (Table S12†) for all *S* = 5/2 models range between 1.87 and 2.20 MHz, also aligning with experimental results. It is noteworthy that the nuclear quadrupole asymmetry parameter (*η*) has been a defining criterion in other contexts, such as ammonia binding on the OEC,^86^ but in this case only the *g*_*x*_ component of the ESEEM spectra was detectable,^178^ thus no information on *η* could be derived. Based on our calculations, high anisotropy is predicted for the ^14^N NQI tensors of all high-spin models, ranging between 0.73 and 0.97, except for models D2 and D6, which show values of 0.57 and 0.58, respectively. Therefore, all proposed *S* = 5/2 models regardless of their chemical type have similar predicted ^14^N HFCs, NQI constants, and *η* values, and are consistent with ESEEM experimental data for the *g* = 4.1 EPR signal. Thus any attempt to distinguish between these *S* = 5/2 models based on the ESEEM data is misguided.

**Table 7:** Calculated and experimental His332 ^14^N projected isotropic hyperfine coupling constants (MHz) in the low- and high-spin variations

	|*A*_iso_|, MHz	|*a*_iso_|, MHz
A1	5.8	3.1
A2	5.6	3.0
*Synechocystis* ^86^	7.2	
*T. vestitus* ^85^	7.1	
B1	0.9	1.8
B2	0.9	1.7
B3	0.6	1.1
C1	0.9	3.3
C2	0.9	3.1
C3	0.9	3.1
C4	0.8	2.8
D1	0.8	1.9
D2	0.5	0.9
D4	0.9	1.9
D5	0.7	1.7
D6	0.6	1.4
D7	0.9	1.5
*Spinach* ^178^	1	

We now examine the electronic structure basis for the similarity between the ^14^N HFCs of all high-spin models. The value of *A*_iso_ for a ligand nitrogen atom depends on the degree of overlap between the nitrogen nucleus and the Mn 3d orbitals, which contain the unpaired electrons, and on the projection factor (*ρ*_*i*_) for the coordinating Mn ion. In all S_2_ models, the His332 N atom is coordinated either in the equatorial position to an axially elongated Mn1(iii) ion with a (3d_*xy*_,3d_*xz*_,3d_*yz*_)^3^(3d_*z*^2^_)^1^ electron configuration or to a pseudo-octahedral Mn1(iv) ion with a (3d_*xy*_,3d_*xz*_,3d_*yz*_)^3^ electron configuration. Therefore, the nitrogen nucleus interacts with the empty d_*x*^2^−*y*^2^_ or d_*z*^2^_ orbital of Mn1. The intrinsic (on-site) ^14^N *α*_iso_ values of all models, which are given in Table 7, fall in the range 0.9–3.3 MHz, close to the values reported for model complexes in which histidine is coordinated to the equatorial position of Mn(iii), or to the equatorial or axial position of Mn(iv), *i.e.* 2.2–2.9 MHz.^190^ More specifically, the intrinsic *α*_iso_ values for all ^14^N coordinated to the equatorial position of Mn(iii) range between 2.8 and 3.1 MHz, except from model D2 with ^14^N *α*_iso_ 0.9 MHz, probably due to the electron-withdrawing effect of the OH ligand on Mn1. In contrast, the intrinsic *α*_iso_ values for all ^14^N coordinated to Mn1(iv) are in the range 1.1–1.9 MHz. The spin-projection factor (*ρ*_*i*_) of a spin center *i* is defined as the ratio of the spin expectation value 〈*S*_*z*_^*i*^〉 on Mn_*i*_ to the total spin of the cluster. The calculated spin expectation values and spin-projection factors of all Mn ions are given in Tables S13 and S14,† respectively. The spin projection factors of Mn1 are smaller than 0.6 for all high-spin models, whereas for the low-spin models they are ∼1.6. Hence, the small ^14^N *A*_iso_ for His332 in all high-spin models is attributed to the higher total spin of the cluster.

^55^Mn HFCs have not been experimentally determined for high-spin S_2_ signals. Nevertheless, addition of NH_4_Cl in spinach S_2_ state samples that contain the *g* ≈ 4.1 signal reveals hyperfine structure with a regular spacing of 36 G.^191,192^ Under these conditions, ammonia binds proximal to the cluster, possibly near the proximal Cl^−^.^193^ The narrowing of the spacing between the peaks of the hyperfine structure relative to the low-spin multiline signal, *i.e.* 16–42 G in the high-spin *vs.* 60–90 G in the low-spin, suggests that the Mn HFCs in the high-spin state are 40–70% relative to those of the low-spin form.^178^ Moreover, extrinsic protein-depleted PSII in the presence of large concentrations of Ca^2+^ ions generates a *g* = 4.86 EPR signal^68^ that was assumed to be related to the *g* ≈ 5 signal arising from near-infrared illumination or decay of the S_3_ state at cryogenic temperatures.^97,98^ This signal exhibits hyperfine structure reproduced by a set of four isotropic hyperfine splittings between 14 and 26 G.^68^ Here, we calculated the ^55^Mn HFCs of all models, and the results, which are given in Tables S15–S17,† are in line with these ranges. However, more reliable ^55^Mn hyperfine values obtained by ENDOR would be required to potentially enable differentiation between models based on calculated hyperfine data. Overall, neither the ^14^N nor the ^55^Mn HFCs allow reliable exclusion of possible models based on experimental observations for the high-spin EPR signals.

### 3.6. Mn K pre-edge X-ray absorption spectroscopy

Mn K-edge X-ray absorption spectroscopy offers complementary insights into the cluster's electronic structure and has been used for the characterization of all spectroscopically observable states of the S-state cycle.^171,194–196^ In a Mn K-edge X-ray absorption spectrum, the onset of absorption involves predominantly transitions from the Mn 1s core electrons to Mn d-orbitals. Although these transitions are dipole-forbidden, they gain intensity due to p–d orbital mixing, which arises from deviations of the metal ion symmetry from centrosymmetry.^197^ This region, known as the pre-edge, precedes the edge region, where dipole-allowed excitations from the Mn 1s electrons to unoccupied molecular orbitals occur, resulting in significantly higher intensity. The edge energy is correlated with the sum of Mn oxidation states of the cluster and its derivative shape could reflect major geometry changes in the Mn coordination sphere. Notably, Liang *et al.*^103^ reported that in samples exhibiting the *g* ≈ 4.1 signal the edge shape changes relative to the low-spin form.

The pre-edge region reveals information on the local electronic structure and coordination geometry of the Mn ions. We recently showed that the pre-edge region combined with QM calculations could clearly discriminate between different S-state isomers, including different oxidation states, protonation states, and Jahn–Teller isomerism.^196^ Consequently, the pre-edge region could, in principle, be used to distinguish between the possible high-spin models, all of which have the same Mn oxidation states but distinct geometries and, in some cases, distinct valence distributions. To investigate whether the pre-edge XAS region can be employed as an additional criterion to elucidate the structural origins of the high-spin models, we calculated the Mn XAS pre-edge region of each model.

**Fig. 7:**
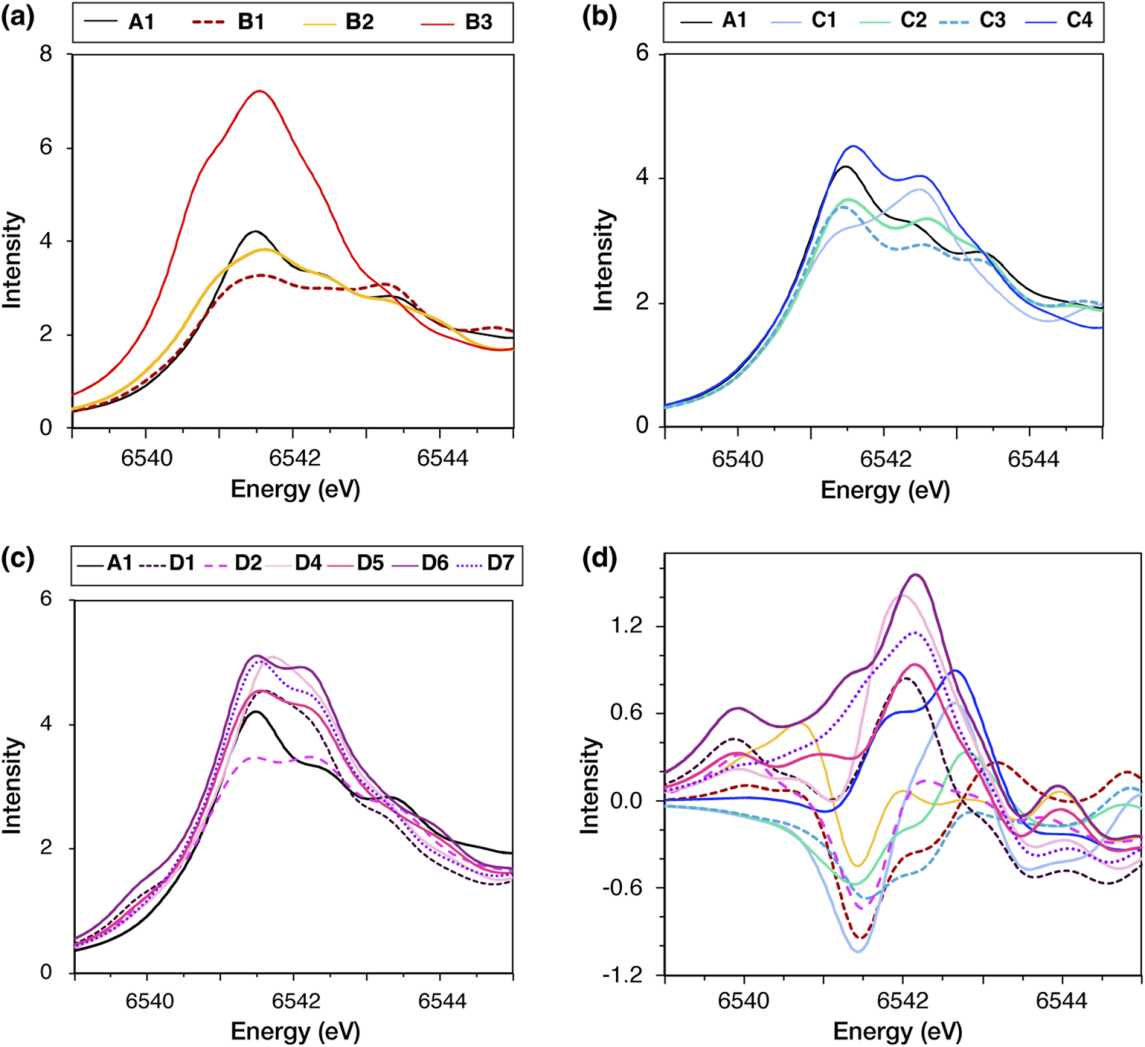
Calculated Mn K pre-edge XAS spectra of the OEC for the high-spin S_2_ state variants of types (a) B, (b) C, and (c) D, compared to the low-spin form A1, shown in black in each plot. Panel (d) shows the difference spectra of each high-spin model from A1, *i.e.* high-spin–low-spin.

The calculated spectra of all high-spin models compared to the low-spin model A1 are shown in Fig. 7a–c. The pre-edge regions of all models feature a broad peak in the region 6541–6542 eV, as well as two higher energy and lower intensity broad peaks between 6542 eV and 6544 eV. Only in models B1, D2, and C1 the intensity of the higher energy features is similar to (B1, D2) or greater than (C1) the lower energy peak. The difference spectra of the high-spin models relative to A1, plotted in Fig. 7d, show that the models can be distinguished into two groups based on the absorption intensity in the region between 6541 and 6543 eV. In this region, models C1, D1, D4, D5, D6, and D7 exhibit more intense absorption than A1, whereas B1, C2, C3, and D2 exhibit lower intensity. Since all models have the same Mn oxidation states, *i.e.* three Mn(iv) and one Mn(iii) ions, the differences in the pre-edge features are due to differences in the local Mn geometry of each model. Model B3 exhibits significantly higher absorption intensity than all other S_2_ models, because its Mn4(iv) ion is significantly distorted from the octahedral geometry.

These results suggest that distinguishing between most models based on the pre-edge region of Mn XAS may be possible if the experimental data will have sufficiently high resolution. Regarding available experimental data, Chatterjee *et al.*^104^ reported that Mn XAS spectra for the low-spin and high-spin forms of the S_2_ state are similar, with a unique difference in the intensity of the lower energy peak in the pre-edge region (∼6541 eV) being slightly less for the high-spin isomer. Higher-resolution methods, such as high energy resolution fluorescence detected (HERFD) X-ray spectroscopy, could provide more confident distinction. Nevertheless, the models most consistent with the experimental data presently available are B1, B2, C2, C3, and D2, of which only B1, B2, and D2 may be energetically plausible as discussed above.

## 4. Conclusions and outlook

The S_2_ state of the OEC exhibits a range of different EPR signals at *g* values higher than the low-spin multiline signal between different organisms and under various treatments. Elucidation of the structural origins of these high-spin forms is important not only to determine the nature of the S_2_ state but also to get insight into the mechanism of the S_2_ → S_3_ transition that precedes O–O bond formation. Previous computational studies have conceptualized three ideas about the molecular frameworks that can give rise to high-spin ground states and may thus be responsible for these signals. These are the open/closed cubane valence isomerism hypothesis (models of type B in the present work),^99^ O4 oxo-bridge protonation (models of type C),^102^ and early water binding to the OEC cluster in the S_2_ state (models of type D).^101^

We incorporated all these ideas in quantum chemical modeling to compare their structures and properties on an equal basis, using the largest quantum chemical models ever reported for the OEC, exceeding 2000 atoms. For this purpose, we defined a two-layer QM approach combining DFT with an extended semiempirical tight-binding model (xTB) that enables the explicit QM treatment of very large structural models. The DFT/xTB approach demonstrates smooth convergence of structural, magnetic, and spectroscopic parameters with respect to the size of the QM region, while providing reliable QM/xTB relative energies. While previous studies have demonstrated the applicability of the highly accurate coupled-cluster methods on OEC isolated clusters for relative energy calculations,^198–202^ we here applied for the first time a DLPNO-CCSD(T)/xTB protocol. This approach combines a high-level correlated method for the metallocofactor with a semiempirical quantum chemical method for a very large part of its environment, setting the stage for unprecedently high-accuracy calculations on metalloenzymes. Overall, our findings suggest QM/xTB as a powerful method for studying metalloproteins, offering significant practical advantages over conventional QM/MM methods for geometry optimizations and property calculations.

We first addressed whether it is possible to differentiate between the three ideas by using the best possible models and refined approaches in terms of their total spin. It appears that this is not possible, as there are several models with predicted *S* = 5/2 and *S* = 7/2 ground spin states, which are associated with at least two of the observed high-spin EPR signals, with *g* = 4.1 and *g* = 4.75. Nevertheless, our results constrain the possible scenarios about the structural origins of the high-spin signals to different valence isomers or protonation states of early water binding forms or to a mixture of models that represent different ideas. Energetic considerations are crucial because they show protonation of O4 to be practically impossible, thereby invalidating models of type C as candidates for the high-spin forms of the S_2_ state. This is supported by two-layer DLPNO-CCSD(T)/xTB calculations applied for the first time in this type of system. Instead, the present results still allow for a combination or mixture of B and D type models, while the B-type valence isomeric forms have the advantage of being energetically very close to the low-spin ground state.

Other experimentally observable properties that could be used to further differentiate between the models were also considered. We show that neither the ^14^N nor ^55^Mn HFCs can in principle distinguish between the models. However, Mn K-pre-edge features could indeed be used, provided that the pre-edge region will be sufficiently resolved and that homogeneous samples of specific high-spin forms can be prepared.

In addition to the structural origins of each high-spin S_2_ state signal, it is of fundamental interest to understand the effect of different chemical treatments and experimental conditions on the structure of PSII and to elucidate how specific chemical changes at the OEC map to EPR observables. A central computational challenge is the accurate determination of acidities in order to explain the pH effects on the EPR signals of the OEC.^19,93^ Description of these effects may require going beyond static approaches^203^ and using molecular dynamics to account for changes in the hydrogen bonding networks that might extend relatively far from the OEC cluster.^36^ Similar requirements are related to the description of effects that result from mutations,^70–77^ cryoprotectants, and treatments targeting extrinsic proteins.^67–69^ These effects might not be proximal to the Mn cluster but influence its properties exclusively through second-sphere changes. The restricted size of current computational models might place such differences outside the model limits. Therefore, a very important future target for computational modeling is to account for long-range effects by explicit treatment of a much larger part of the protein around the OEC cluster. Our models and the two-level QM computational approach described in this work are useful in all these directions, particularly if they are combined in the future with *ab initio* molecular dynamics and with an outer molecular mechanics layer to incorporate long-range inhomogeneous electrostatic effects.

## Author contributions

M. A. M.: investigation, analysis, writing – original draft; M. D.: methodology, investigation, analysis, writing – original draft; D. A. P.: conceptualization, methodology, supervision, writing – review and editing.

## Conflicts of interest

There are no conflicts to declare.

## Supplementary Material

SC-016-D4SC07818G-s001

## Data Availability

Data supporting this article have been included as part of the ESI;† complete computational models, input files for the multilayer quantum chemical optimizations, and results of TD-DFT calculations are available at Edmond, the Open Research Data Repository of the Max Planck Society, at https://doi.org/10.17617/3.YVJ4UJ.
