# Supplementary material for: On the nature of high-spin forms in the S2 state of the oxygen-evolving complex
Source: Chem Sci. 2025 Jan 31;16(9):4023–47. doi: 10.1039/d4sc07818g (PMC11784572; doi:10.1039/d4sc07818g)
Supplement: SC-016-D4SC07818G-s001 [file SC-016-D4SC07818G-s001.pdf]

Supplementary Information (SI) for Chemical Science.
This journal is © The Royal Society of Chemistry 2025

On the Nature of High-Spin Forms in the S_2 State of the Oxygen-Evolving Complex

Markella Aliki Mermigki, Maria Drosou, Dimitrios A. Pantazis[*]

Max-Planck-Institut für Kohlenforschung, Kaiser-Wilhelm-Platz 1, 45470 Mülheim an der Ruhr, Germany

ELECTRONIC SUPPLEMENTARY INFORMATION

Table S1. Selected interatomic distances (Å) for the set of optimizations where the QM1 region was optimized while keeping all atoms of the QM2 region constrained.

	Mn1-Mn2	Mn2-Mn3	Mn3-Mn4	Mn1-Mn3	Mn1-Mn4	Mn1-Ca	Mn2-Ca	Mn3-Ca	Mn4-Ca
**Q0**	2.741	2.733	2.710	3.248	4.851	3.497	3.316	3.536	4.002
**Q1**	2.729	2.719	2.705	3.213	4.737	3.519	3.339	3.513	3.885
**Q2**	2.733	2.708	2.702	3.215	4.733	3.506	3.414	3.627	3.960
**Q3**	2.734	2.717	2.697	3.235	4.778	3.474	3.403	3.590	3.906
**Q4**	2.733	2.718	2.699	3.238	4.775	3.472	3.404	3.588	3.892
**Q5**	2.731	2.717	2.700	3.239	4.765	3.474	3.401	3.597	3.895

Table S2. Selected interatomic distances (Å) for the set of optimizations where both the QM1 region and the structurally active part of the QM2 region (605 atoms in total) were simultaneously optimized.

	Mn1-Mn2	Mn2-Mn3	Mn3-Mn4	Mn1-Mn3	Mn1-Mn4	Mn1-Ca	Mn2-Ca	Mn3-Ca	Mn4-Ca
**Q0**	2.716	2.727	2.696	3.209	4.716	3.478	3.290	3.535	3.919
**Q1**	2.723	2.722	2.700	3.198	4.708	3.513	3.346	3.556	3.883
**Q2**	2.732	2.717	2.698	3.200	4.703	3.482	3.394	3.624	3.908
**Q3**	2.730	2.723	2.700	3.216	4.766	3.462	3.383	3.577	3.867
**Q4**	2.729	2.723	2.701	3.221	4.767	3.458	3.385	3.579	3.861
**Q5**	2.731	2.723	2.702	3.224	4.759	3.456	3.393	3.593	3.868

Table S3. Mulliken spin populations on Mn ions for the set of optimizations where the QM1 region was optimized while keeping all atoms of the QM2 region constrained.

	Mn1	Mn2	Mn3	Mn4
**Q0**	3.92	2.97	2.94	2.91
**Q1**	3.91	2.95	2.94	2.95
**Q2**	3.90	2.95	2.93	2.96
**Q3**	3.91	2.96	2.95	2.96
**Q4**	3.91	2.96	2.95	2.96
**Q5**	3.90	2.96	2.96	2.96

Table S4. Mulliken spin populations on Mn ions for the set of optimizations where both the QM1 region and the structurally active part of the QM2 region were simultaneously optimized.

	Mn1	Mn2	Mn3	Mn4
**Q0**	3.89	2.96	2.94	2.91
**Q1**	3.88	2.95	2.95	2.95
**Q2**	3.88	2.95	2.94	2.96
**Q3**	3.89	2.95	2.96	2.95
**Q4**	3.89	2.95	2.96	2.96
**Q5**	3.89	2.95	2.96	2.95

Table S5. BS-TPSSh calculated pairwise Mn-Mn exchange coupling constants J_{ij} (cm^{-1}) and energy differences between the ground and the first excited state (cm^{-1}) for the set of optimizations where the QM1 region was optimized while keeping all atoms of the QM2 region constrained.

	J_{12}	J_{13}	J_{14}	J_{23}	J_{24}	J_{34}	$\Delta E_{ES\text{-}GS}$
**Q0**	-23	0	9	16	3	-28	41.3
**Q1**	-21	1	9	20	3	-15	26.9
**Q2**	-21	0	8	15	2	-15	28.3
**Q3**	-21	2	7	17	2	-13	24.0
**Q4**	-21	2	7	17	2	-13	24.0
**Q5**	-21	2	7	17	2	-13	24.0

Table S6. BS-TPSSh/xTB calculated exchange coupling constants J_{ij} (cm^{-1}) and energy differences between the ground and the first excited state (cm^{-1}) for the set of optimizations where the QM1 region was optimized while keeping all atoms of the QM2 region constrained.

	J_{12}	J_{13}	J_{14}	J_{23}	J_{24}	J_{34}	$\Delta E_{ES\text{-}GS}$
**Q0**	-22	-1	8	15	3	-21	35.7
**Q1**	-22	1	10	18	3	-14	26.4
**Q2**	-22	0	10	15	2	-14	27.1
**Q3**	-21	1	8	16	2	-13	25.0
**Q4**	-21	1	8	16	2	-13	25.7
**Q5**	-21	2	9	16	2	-13	24.2

Table S7. BS-TPSSh/xTB calculated exchange coupling constants J_{ij} (cm^{-1}) and energy differences between the ground and the first excited state (cm^{-1}) for the set of optimizations where both the QM1 region and the structurally active part of the QM2 region were simultaneously optimized.

	J_{12}	J_{13}	J_{14}	J_{23}	J_{24}	J_{34}	$\Delta E_{ES\text{-}GS}$
**Q0**	-23	1	15	19	3	-19	32.1
**Q1**	-18	0	9	25	6	-18	25.5
**Q2**	-20	2	12	18	2	-13	23.6
**Q3**	-21	1	9	17	2	-12	23.4
**Q4**	-22	1	10	17	3	-12	23.6
**Q5**	-21	2	10	17	2	-10	21.0

Table S8. Computed "raw" hyperfine coupling constants $A_{iso,BS}$ (MHz) for all four Mn ions for the set of optimizations where the QM1 region was optimized while keeping all atoms of the QM2 region constrained.

	Mn1	Mn2	Mn3	Mn4
**Q0**	-399	406	427	-462
**Q1**	-426	416	425	-477
**Q2**	-430	415	425	-477
**Q3**	-436	412	419	-477
**Q4**	-436	412	419	-475
**Q5**	-433	411	420	-473

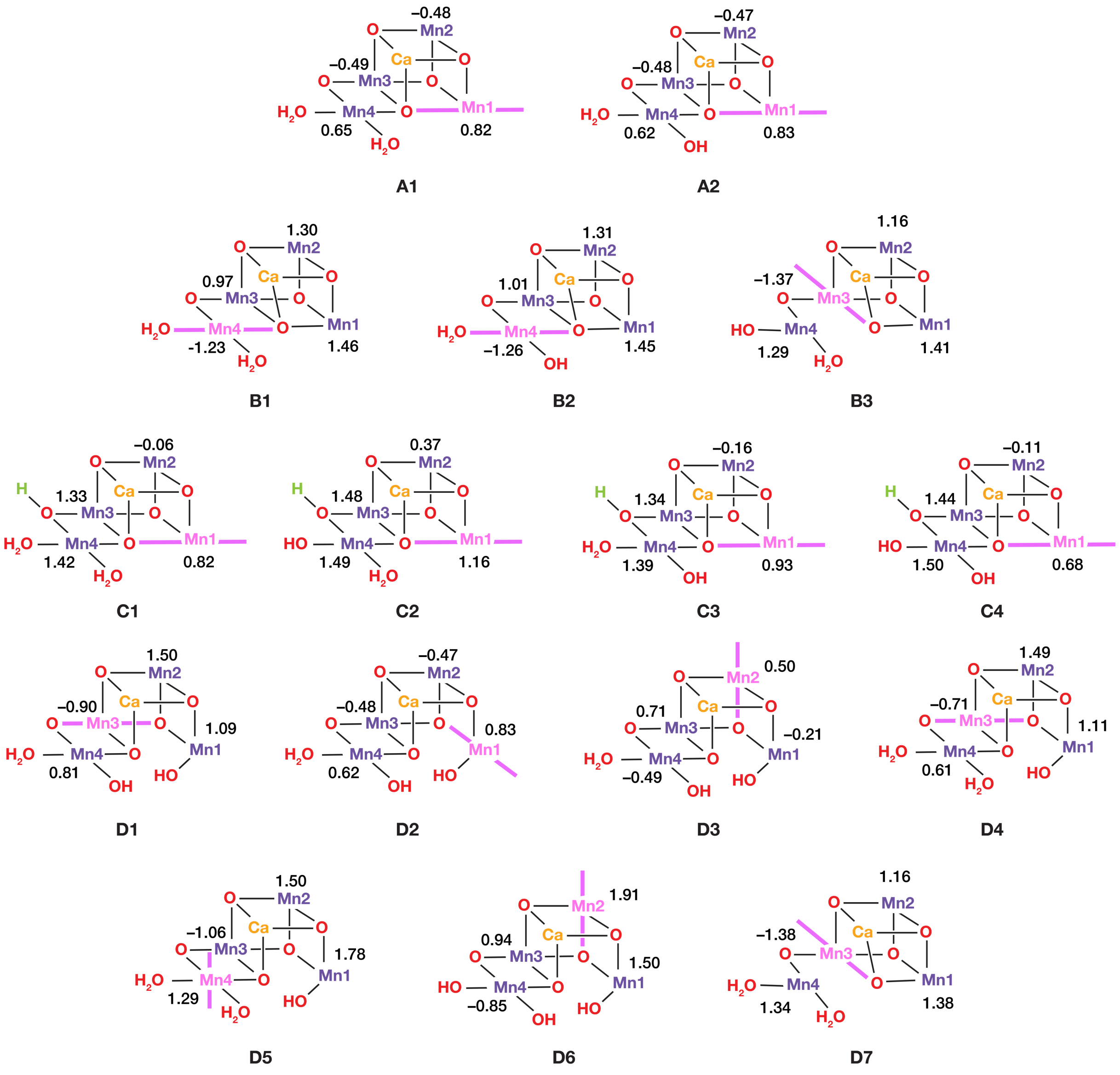

Figure S1. Structure of all S_2 models with the expectation values of the site-spin operators of each Mn ion i, $< S_z^i >$. The Mn(III) ions are shown in pink and Mn(IV) ions in purple. The pseudo-Jahn–Teller axes of Mn(III) ions are indicated with bold pink lines.

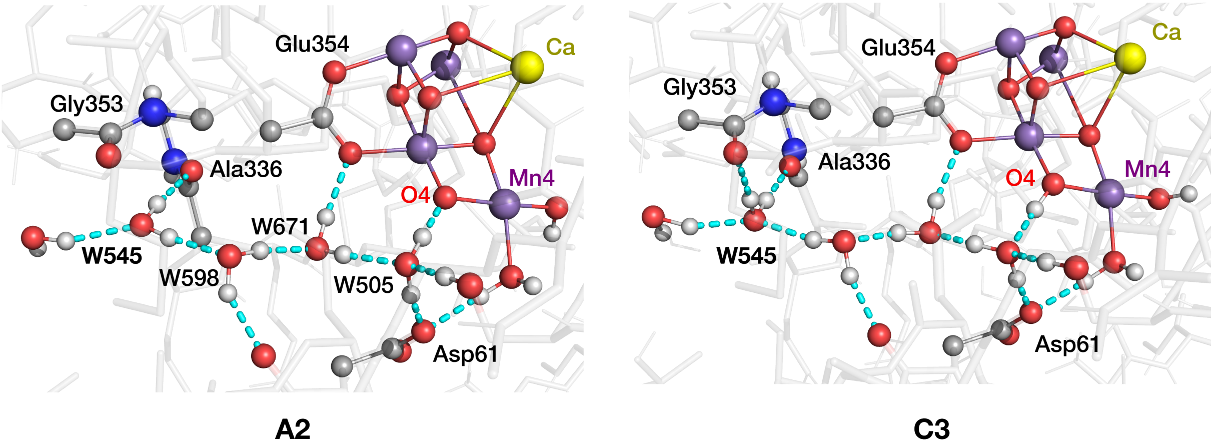

Figure S2. Comparison of the hydrogen bonding networks of the low-spin model **A2** and the high-spin model **C3** with protonated O4 oxo-bridge showing the differences in the O4 channel hydrogen bonds. When O4 is not protonated, the crystallographic water molecule W505 forms a hydrogen bond with O4 and with the carbonyl oxygen of Asp61. Moreover, W545 forms a hydrogen bond with the carbonyl oxygen of Ala336 and is close (3.2 Å) to Gly353 without forming a hydrogen bond. Upon O4 protonation, the direction of the W505 proton changes from O4 to W671. In response to this, the directions of the protons connecting water molecules W545 and W598 are expected to change as well, and W545 can now form hydrogen bonds with the carbonyl oxygen atoms of both Ala336 and Gly353.

Table S9. Mulliken spin populations (TPSSh) on the Mn ions of all optimized S_2 state models.

	Mn1	Mn2	Mn3	Mn4
**A1**	3.910	2.960	2.957	2.956
**A2**	3.902	2.937	2.902	2.928
**B1**	2.961	2.921	2.905	3.948
**B2**	2.971	2.912	2.882	3.897
**B3**	2.954	2.934	3.763	2.922
**C1**	3.924	2.964	2.987	2.989
**C2**	3.930	2.948	2.934	2.906
**C3**	3.926	2.956	2.973	2.947
**C4**	3.917	2.924	2.936	2.984
**D1**	2.974	3.023	3.795	2.926
**D2**	3.883	2.985	2.961	2.926
**D3**	2.965	3.863	2.944	2.923
**D4**	2.965	3.863	2.944	2.923
**D5**	3.033	2.977	3.816	2.882
**D6**	2.928	2.948	2.906	3.875
**D7**	3.032	2.923	3.795	2.908

Table S10. Relative energies (in kcal/mol) per group of isomers of the S_2 variants, calculated with B3LYP/xTB and DLPNO-CCSD(T)/xTB using the "QM1" region of ca. 130 atoms defined in Section 2.2, as well as the "QM4" region of ca. 260 atoms using different functionals (B3LYP, B3LYP* and TPSSh). For each methodology the energy differences of both QM/xTB and isolated QM are noted.

	B3LYP (Q1)		DLPNO (Q1)		B3LYP (Q4)		B3LYP* (Q4)		TPSSh (Q4)	
	QM/ xTB	QM	QM/ xTB	QM	QM/ xTB	QM	QM/ xTB	QM	QM/ xTB	QM
**A1**	0.0	0.0	0.0	0.0	0.0	0.0	0.0	0.0	0.0	0.0
**B1**	3.4	1.5	-1.2	-2.9	5.5	2.6	5.6	2.7	4.9	2.0
**C2**	33.0	24.3	35.7	27.1	32.4	21.6	32.5	21.7	32.3	21.5
**C3**	24.8	17.1	30.2	22.5	24.2	8.1	24.3	8.1	24.9	8.7
**A2**	0.0	0.0	0.0	0.0	0.0	0.0	0.0	0.0	0.0	0.0
**B2**	-1.0	0.5	-6.6	-5.0	1.6	3.7	1.3	3.4	0.9	3.1
**B3**	2.2	21.5	-3.2	16.1	2.6	23.6	2.4	23.3	2.0	23.0
**C4**	29.6	28.6	31.1	30.2	30.9	16.9	31.7	17.7	30.2	16.2

Table S11. Calculated exchange coupling constants J_{ij} (cm^{-1}), ground spin state (S_{GS}), first excited spin state (S_{ES}), and their energy separation $\Delta E_{GS–ES}$ (cm^{-1}) for selected models of the S_2 state with protonated and neutral His337.

		J_{12}	J_{13}	J_{14}	J_{23}	J_{24}	J_{34}	S_{GS}	S_{ES}	$\Delta E_{ES–GS}$
**A2**	**His337^{+1}**	-27	4	8	13	2	-12	1/2	3/2	24.1
	**His337^{0}**	-28	4	9	14	3	-13	1/2	3/2	24.0
**B2**	**His337^{+1}**	27	11	9	27	1	-22	5/2	7/2	3.6
	**His337^{0}**	27	8	13	28	1	-20	7/2	5/2	10.0
**C4**	**His337^{+1}**	-28	17	4	26	0	9	7/2	9/2	12.4
	**His337^{0}**	-31	19	5	28	1	10	7/2	9/2	13.4
**D1**	**His337^{+1}**	19	-39	-3	14	6	-51	5/2	3/2	53.1
	**His337^{0}**	19	-36	-2	10	5	-49	5/2	3/2	71.3

Table S12. Calculated His332 ^{14}N unprojected ("raw") isotropic hyperfine coupling constants ($A_{BS,iso}$ MHz), the site isotropic coupling constant ($a_{iso,site}$ MHz) and the Anisotropy of the NQI Tensors (MHz).

	$\|A_{BS,iso}\|$	$\|a_{iso}\|^1$	$\|e^{2Qq/h}\|$	η
**A1**	12.5	3.1	2.04	0.99
**A2**	11.8	3.0	2.12	0.60
**B1**	1.1	1.8	1.87	0.94
**B2**	1.0	1.7	1.95	0.93
**B3**	0.7	1.1	2.09	0.82
**C1**	13.1	3.3	1.99	0.88
**C2**	1.7	3.1	2.04	0.97
**C3**	1.8	3.1	1.98	0.97
**C4**	1.6	2.8	2.09	0.89
**D1**	1.2	1.9	2.20	0.73
**D2**	0.5	0.9	2.49	0.57
**D4**	1.2	1.9	2.05	0.85
**D5**	0.7	1.7	1.98	0.89
**D6**	0.6	1.4	2.42	0.58
**D7**	0.9	1.5	2.07	0.85

[1] $|a_{iso}| = |A_{BS,iso}| \cdot \frac{\langle S_z \rangle_{BS}}{S_i}$ where $|A_{BS,iso}|$ is the raw calculated hyperfine coupling constant, $\langle S_z \rangle_{BS}$ is the total spin M_S of the BS determinant and S_i the site-spin of the coordinating Mn ion.

Table S13. BS-DFT calculated spin expectation values $< S_z^i >$ for the Mn ions of all S_2 state models.

	Mn1	Mn2	Mn3	Mn4
**A1**	0.822	-0.477	-0.490	0.646
**A2**	0.832	-0.471	-0.477	0.616
**B1**	1.460	1.298	0.970	-1.229
**B2**	1.451	1.305	1.005	-1.261
**B3**	1.414	1.164	-1.369	1.291
**C1**	0.822	-0.064	1.326	1.416
**C2**	1.162	0.369	1.479	1.490
**C3**	0.896	-0.182	1.367	1.419
**C4**	0.888	-0.322	1.455	1.478
**D1**	1.085	1.499	-0.895	0.811
**D2**	1.780	1.495	-1.062	1.287
**D4**	1.112	1.493	-0.713	0.609
**D5**	1.495	1.500	-0.588	1.093
**D6**	1.499	1.911	0.943	-0.853
**D7**	1.380	1.164	-1.384	1.341

Table S14. Spin-projection factors (ρ_i) of all Mn ions of the S_2 state models.

	Mn1	Mn2	Mn3	Mn4
**A1**	1.644	-0.955	-0.981	1.292
**A2**	1.663	-0.942	-0.954	1.233
**B1**	0.584	0.519	0.388	-0.492
**B2**	0.580	0.522	0.402	-0.504
**B3**	0.566	0.466	-0.548	0.517
**C1**	0.234	-0.017	0.379	0.406
**C2**	0.258	0.082	0.255	0.331
**C3**	0.256	-0.052	0.391	0.405
**C4**	0.254	-0.092	0.416	0.422
**D1**	0.432	0.600	-0.356	0.324
**D2**	0.509	0.426	-0.303	0.366
**D4**	0.445	0.597	-0.285	0.244
**D5**	0.427	0.428	-0.168	0.312
**D6**	0.429	0.546	0.269	-0.243
**D7**	0.552	0.465	-0.554	0.536

Table S15. Raw calculated hyperfine coupling constants $A_{iso,BS}$ (MHz) for all four Mn ions for all high-spin variations.

	Mn1	Mn2	Mn3	Mn4
**A1**	-441	410	416	-477
**A2**	-455	428	377	-472
**B1**	-65	-65	-62	81
**B2**	-65	-66	-60	92
**B3**	-72	-76	132	-78
**C1**	-420	391	400	-460
**C2**	-57	70	-62	-58
**C3**	-56	70	-60	-55
**C4**	-69	70	-63	-59
**D1**	119	-94	119	-94
**D2**	-70	-62	69	-68
**D4**	-69	-74	124	-90
**D5**	-51	-54	68	-97
**D6**	70	-69	-50	70
**D7**	-71	-75	133	-84

Table S16. Local isotropic ^{55}Mn hyperfine coupling constants a_{iso} (MHz) for all S_2 models.

	$a_{1,iso}$	$a_{2,iso}$	$a_{3,iso}$	$a_{4,iso}$
**A1**	-196	243	247	-283
**A2**	-203	254	224	-280
**B1**	-194	-194	-184	181
**B2**	-192	-195	-178	205
**B3**	-213	-225	294	-230
**C1**	-187	232	237	-273
**C2**	-177	292	-259	-241
**C3**	-190	237	223	-268
**C4**	241	-241	-212	-245
**D1**	352	-278	264	-278
**D2**	-217	-257	287	-281
**D4**	-204	-221	276	-267
**D5**	-210	-225	283	-304
**D6**	291	-214	-208	291
**D7**	-212	-223	296	-249

Table S17. Experimental and calculated ^{55}Mn projected and scaled isotropic hyperfine coupling constants ($|A_{iso}|$, in MHz) for all S_2 models arranged in the descending order ($A_1 > A_2 > A_3 > A_4$) with the corresponding Mn ion indicated in square brackets, and the ratios A_1/A_2, A_2/A_3, A_3/A_4.

		$\|A_{1,iso}\|$	$\|A_{2,iso}\|$	$\|A_{3,iso}\|$	$\|A_{4,iso}\|$	A_1/A_2	A_2/A_3	A_3/A_4
Calculation	**A1**	366 [4]	322 [1]	242 [3]	232 [2]	1.14	1.33	1.04
	**A2**	345 [4]	337 [1]	239 [2]	213 [3]	1.02	1.41	1.12
Experiment	*Synechocystis*[1]	313	302	246	210	1.04	1.23	1.17
	Spinach[1]	368	284	244	234	1.30	1.16	1.04
	T. vestitus[2]	307	209	204	190	1.47	1.02	1.07
Calculation	**B1**	113 [1]	101	89 [4]	71 [3]	1.12	1.13	1.24
	**B2**	112 [1]	103 [4]	102 [2]	72 [3]	1.08	1.02	1.42
	**B3**	161 [3]	120 [1]	119 [4]	105 [2]	1.34	1.01	1.13
	**C1**	111 [4]	90 [3]	44 [1]	4 [2]	1.23	2.06	11.01
	**C2**	80 [4]	66 [3]	46 [1]	24 [2]	1.20	1.45	1.90
	**C3**	98 [3]	92 [4]	44 [1]	15 [2]	1.06	2.08	2.94
	**C4**	109 [3]	103 [4]	54 [1]	27 [2]	1.05	1.91	2.02
	**D1**	167 [2]	152 [1]	94 [3]	90 [4]	1.10	1.62	1.04
	**D2**	111 [1]	109 [2]	103 [4]	87 [3]	1.01	1.06	1.18
	**D4**	132 [2]	91 [1]	78 [3]	64 [4]	1.45	1.16	1.22
	**D5**	96 [2]	95 [4]	90 [1]	48 [3]	1.02	1.06	1.88
	**D6**	125 [1]	117 [2]	71 [4]	56 [3]	1.07	1.65	1.26
	**D7**	164 [3]	134 [4]	117 [1]	104 [2]	1.23	1.14	1.13

The ^{55}Mn projected and scaled isotropic hyperfine coupling constants are calculated using the well-established procedure:

$$|A_{i,\mathrm{iso}}| = \left|\frac{A_{i,x} + A_{i,y} + A_{i,z}}{3}\right|$$

Where $A_{i,x} = A_{x,\mathrm{BS}} \cdot \frac{\langle S_z \rangle_{\mathrm{BS}}}{S_i} \cdot \rho_i \cdot 1.78$, and same for $A_{i,y}$ and $A_{i,z}$.

$A_{x,\mathrm{BS}}$ is the raw calculated Hyperfine Coupling Constant, $\langle S_z \rangle_{\mathrm{BS}}$ is the total spin M_S of the BS determinant, ρ_i is the spin projection coefficient of Mn_i and 1.78 is a scaling factor used for comparison of the calculated ^{55}Mn hyperfine coupling constants with experimental results.

Orca Input File Examples

DFT/xTB Geometry Optimizations

```
! QM/XTB UKS B3LYP RIJCOSX ZORA ZORA-def2-TZVP(-f) D4 SARC/J
! KDIIS NoTrah TightSCF
! Opt

%pal nprocs 10 end
%maxcore 10000

%basis NewGTO C "ZORA-def2-SVP" end
       NewGTO H "ZORA-def2-SVP" end
end

%qmmm
     QMAtoms {57:68} end
     ActiveAtoms {57:68} end
     Charge_Total 0               # charge of the full system.
     Mult_Total 14                # multiplicity of the full system.
end

%rel OneCenter true
end

%scf maxiter 200
     shift shift 0.10 erroff 0.1 end
     CNVSOSCF true SOSCFstart 0.001
end

*xyzfile 2 14 6dhfAx12A.xyz
```

DLPNO-CCSD(T)/xTB Single Point Calculations

```
! QM/XTB UKS B3LYP DLPNO-CCSD(T1) ZORA ZORA-def2-TZVP def2-TZVP/C decontractAuxC SARC/J
! NoTrah TightSCF NormalPNO
! moread

%pal nprocs 8 end
%maxcore 30000

%moinp "A1_SP_B3LYPXTB.gbw"

%qmmm
     QMAtoms {57:68} end
     Charge_Total 0
     Mult_Total 14
end

%basis newgto H "ZORA-def2-SVP" end
       NewAuxCGTO H "def2-SVP/C" end
       newgto C "ZORA-def2-SVP" end
       NewAuxCGTO C "def2-SVP/C" end
       newgto Mn "ZORA-def2-TZVPP" end
       NewAuxCGTO Mn "def2-TZVPP/C" end
end

%mdci Maxiter 100
end

*xyzfile 1 14 A1.xyz
```

DFT Broken-Symmetry Calculations

```
! UKS TPSSh RIJCOSX ZORA ZORA-def2-TZVP(-f) SARC/J UNO
! Slowconv DEFGRID2 NoTrah TightSCF
! moread

%pal nprocs 10 end
%maxcore 6000
```

```
%moinp "A1_aaaa.gbw"

%basis NewGTO C "ZORA-def2-SVP" end
       NewGTO H "ZORA-def2-SVP" end
end

%cpcm
    surfacetype vdw_gaussian
    epsilon 6.0
end

%scf maxiter 200
     shift shift 0.10 erroff 0.1 end
     FlipSpin 169,170 FinalMs 0.5
end

*xyzfile 2 14 A1.QMRegion.xyz
```

^{55}Mn Hyperfine Coupling Tensors

```
! UKS TPSSh RIJCOSX ZORA ZORA-def2-TZVP(-f) SARC/J
! NoTrah DefGrid3 Slowconv TightSCF
! moread

%pal nprocs 10 end
%maxcore 15000

%moinp "A1_abba.gbw"

%basis newgto C "ZORA-def2-SVP" end
       newgto H "ZORA-def2-SVP" end
       newgto Mn
S 1
   1 4331015.6489100000      1.0000000000
 S 1
   1 1732406.2595600000      1.0000000000
 S 1
   1  692962.5038250000      1.0000000000
 S 1
   1  277185.0015300000      1.0000000000
 S 1
   1   41550.7698900000      1.0000000000
 S 1
   1    9455.9700152000      1.0000000000
 S 1
   1    2676.5206482000      1.0000000000
 S 1
   1     871.4668753000      1.0000000000
 S 1
   1     312.9830642000      1.0000000000
 S 1
   1     121.4445405100      1.0000000000
 S 1
   1      47.9225988300      1.0000000000
 S 1
   1     303.6672316300      1.0000000000
 S 1
   1      93.8814031900      1.0000000000
 S 1
   1      14.8794212100      1.0000000000
 S 1
   1       6.2865200700     -1.0000000000
 S 1
   1       9.4858591300      1.0000000000
 S 1
   1       1.5698706200      1.0000000000
 S 1
   1       0.6590321400     -1.0000000000
 S 1
   1       0.1068629200     -1.0000000000
 S 1
   1       0.0392674400     -1.0000000000
 P 6
   1    1444.7978182000      0.0032493630
   2     342.0655119700      0.0237346473
   3     109.5840089100      0.1067232634
```

```
   4      40.7479881700      0.3007617717
   5      16.1886265700      0.4790147038
   6       6.5484506000      0.2708283465
 P 1
   1      25.3570864400     -1.0000000000
 P 1
   1       3.4830168800      1.0000000000
 P 1
   1       1.3858800900      1.0000000000
 P 1
   1       0.5255509500     -1.0000000000
 P 1
   1       0.1276500000      1.0000000000
 D 4
   1      56.5631891200      0.0192444440
   2      16.2787347100      0.1161060412
   3       5.6964273900      0.3706567953
   4       2.1411147900      0.6570040408
 D 1
   1       0.7829180200      1.0000000000
 D 1
   1       0.2595231100      1.0000000000
 D 1
   1       0.0860000000      1.0000000000
 F 1
   1       1.3260000000      1.0000000000
 end
end

%method SpecialGridAtoms 25
        SpecialGridIntAcc 11
end

%rel picturechange true end

%scf maxiter 200
     shift shift 0.10 erroff 0.1 end
     CNVSOSCF true SOSCFstart 0.001
end

*xyzfile 2 2 A1.QMRegion.xyz

%eprnmr
   nuclei = all Mn { aiso, adip, aorb }
end
```

^{14}N Hyperfine Coupling Tensors

```
! UKS TPSSh RIJCOSX ZORA ZORA-def2-TZVP(-f) SARC/J
! NoTrah DefGrid3 Slowconv TightSCF
! moread

%pal nprocs 10 end
%maxcore 15000

%moinp "A1_abba.gbw"

%basis newgto C "ZORA-def2-SVP" end
       newgto H "ZORA-def2-SVP" end
end

%method SpecialGridAtoms -102
        SpecialGridIntAcc 9
end

%rel picturechange true end

%scf maxiter 200
     shift shift 0.10 erroff 0.1 end
     CNVSOSCF true SOSCFstart 0.001
end

*xyz 2 2
  C   -32.81606358789301     30.28761088251150    361.30983037212343
  C   -33.64819349639328     31.43246819788671    360.71597926986345
  O   -33.04233089349555     32.37744316159161    360.10973068202048
```

```
  ...
  N   -36.39637797714527     37.75964070208878    364.98802961697635 newGTO "EPR-II" end
  H   -40.93938916850582     34.68978276710928    364.09894038433413
  ...
*

%eprnmr
   nuclei = 103 { aiso, adip, aorb, fgrad }
end
```

TD-DFT Calculations of Mn X-Ray Pre-Edge Absorption Spectra

```
! UKS TPSSh RIJCOSX ZORA ZORA-def2-TZVP(-f) SARC/J CPCM
! NoTrah DefGrid3 Slowconv TightSCF
! PrintBasis PrintMOs moread

%pal nprocs 10 end
%maxcore 15000

%moinp "A1_abba.gbw"

%basis newgto C "ZORA-def2-SVP" end
       newgto H "ZORA-def2-SVP" end
end

%cpcm
    surfacetype vdw_gaussian
    epsilon 6.0
end

%tddft NRoots 150
        MaxDim 8
        OrbWin[0]=0,0,-1,-1
        OrbWin[1]=0,0,-1,-1
        DoQuad true
end

*xyzfile 2 2 A1.QMRegion.xyz
```

Broken-Symmetry Total Energies

A1_aaaa:	-12623.696020356470
A1_aaab:	-12623.695992155570
A1_aaba:	-12623.695754559503
A1_aabb:	-12623.694744484541
A1_abaa:	-12623.696423563340
A1_abab:	-12623.696579136809
A1_abba:	-12623.697495624656
A1_baaa:	-12623.696615624282

A2_aaaa:	-12623.231112361276
A2_aaab:	-12623.231065798996
A2_aaba:	-12623.230861661585
A2_aabb:	-12623.229823508897
A2_abaa:	-12623.231928859101
A2_abab:	-12623.232093440551
A2_abba:	-12623.232803980689
A2_baaa:	-12623.231901326084

B1_aaaa:	-12623.697349493696
B1_aaab:	-12623.697850517168
B1_aaba:	-12623.696771192737
B1_abaa:	-12623.694844791593
B1_abba:	-12623.696721762217
B1_baaa:	-12623.695544027962
B1_baba:	-12623.695544316870
B1_bbaa:	-12623.695431961678

B2_aaaa:	-12623.232452545464
B2_aaab:	-12623.233058282956
B2_aaba:	-12623.232061879182
B2_abaa:	-12623.230175463430
B2_abba:	-12623.232000206923
B2_baaa:	-12623.230380141093
B2_baba:	-12623.230930969143
B2_bbaa:	-12623.230321409774

B3_aaaa:	-12623.205548669890
B3_aaab:	-12623.207076268252
B3_aaba:	-12623.210647070171
B3_abaa:	-12623.207216084587
B3_baaa:	-12623.204453615706
B3_baab:	-12623.206206403924
B3_abab:	-12623.208790884171
B3_bbaa:	-12623.209012675627

C1_aaaa:	-12624.107475514689
C1_aaab:	-12624.106615605704
C1_aaba:	-12624.106279355192
C1_aabb:	-12624.106155107949
C1_abaa:	-12624.107702411082
C1_abab:	-12624.106886029955
C1_abba:	-12624.107835830948
C1_baaa:	-12624.107687156158

B2_aaaa:	-12623.670727069777
B2_aaab:	-12623.670146491497
B2_aaba:	-12623.668750740018
B2_aabb:	-12623.668943220111
B2_abaa:	-12623.670856308170
B2_abab:	-12623.670259601962
B2_abba:	-12623.670673881943
B2_baaa:	-12623.670841032519

B3_aaaa:	-12623.676222828066
B3_aaab:	-12623.675617660072
B3_aaba:	-12623.674993793184
B3_aabb:	-12623.674865163957
B3_abaa:	-12623.676566007372
B3_abab:	-12623.676006650330
B3_abba:	-12623.676697302299
B3_baaa:	-12623.676540606522

B4_aaaa:	-12623.194266999557
B4_aaab:	-12623.193648369459
B4_aaba:	-12623.191892057903

```
B4_aabb:     -12623.192002155833
B4_abaa:     -12623.194713312296
B4_abab:     -12623.194124244212
B4_abba:     -12623.194510637717
B4_baaa:     -12623.194643375389

D1_aaaa:     -12699.313915408475
D1_aaab:     -12699.316588063504
D1_aaba:     -12699.318098345284
D1_abaa:     -12699.312167086655
D1_abab:     -12699.315327206981
D1_baaa:     -12699.315379583479
D1_baab:     -12699.317854393790
D1_bbaa:     -12699.315164277492

D2_aaaa:     -12699.319609560973
D2_aaab:     -12699.320584989002
D2_aaba:     -12699.322007824761
D2_aabb:     -12699.320879941788
D2_abaa:     -12699.317827125466
D2_abab:     -12699.318923333873
D2_abba:     -12699.320896870617
D2_baaa:     -12699.319859076842

D3_aaaa:     -12699.315370051121
D3_aaab:     -12699.316099625112
D3_aaba:     -12699.315005636316
D3_aabb:     -12699.314166922721
D3_abaa:     -12699.316229266014
D3_baaa:     -12699.315451084189
D3_baab:     -12699.316624578789
D3_baba:     -12699.316465253214

D4_aaaa:     -12699.792480381404
D4_aaab:     -12699.796262124508
D4_aaba:     -12699.797423689999
D4_abaa:     -12699.790551350978
D4_abab:     -12699.794922687999
D4_baaa:     -12699.793939354609
D4_baab:     -12699.797358087559
D4_bbaa:     -12699.793473845371

D5_aaaa:     -12699.790800352677
D5_aaab:     -12699.798389345880
D5_aaba:     -12699.798704769020
D5_abaa:     -12699.790198613955
D5_abba:     -12699.798486352372
D5_baaa:     -12699.790579628883
D5_baba:     -12699.797838867169
D5_bbaa:     -12699.790771806385

D6_aaaa:     -12698.806495688748
D6_aaab:     -12698.808476539887
D6_aaba:     -12698.807492104392
D6_aabb:     -12698.805538618648
D6_abaa:     -12698.805328651541
D6_baaa:     -12698.806092986049
D6_baab:     -12698.808168353147
D6_baba:     -12698.807146771476

D7_aaaa:     -12699.783200982496
D7_aaab:     -12699.785407407186
D7_aaba:     -12699.789899616864
D7_abaa:     -12699.786037773509
D7_abab:     -12699.787379576335
D7_baaa:     -12699.782988453268
D7_baab:     -12699.785406030851
D7_bbaa:     -12699.788238294081
```

References

1. D. A. Marchiori, P. H. Oyala, R. J. Debus, T. A. Stich and R. D. Britt, Structural Effects of Ammonia Binding to the Mn_4CaO_5 Cluster of Photosystem II, *J. Phys. Chem. B*, 2018, **122**, 1588-1599.
2. T. Lohmiller, V. Krewald, M. P. Navarro, M. Retegan, L. Rapatskiy, M. M. Nowaczyk, A. Boussac, F. Neese, W. Lubitz, D. A. Pantazis and N. Cox, Structure, ligands and substrate coordination of the oxygen-evolving complex of photosystem II in the S_2 state: a combined EPR and DFT study, *Phys. Chem. Chem. Phys.*, 2014, **16**, 11877-11892.
